# Comparison of discovery rates and prognostic utility of [^68^Ga]Ga-PSMA-11 PET/CT and circulating tumor DNA in prostate cancer—a cross-sectional study

**DOI:** 10.1007/s00259-024-06698-7

**Published:** 2024-05-02

**Authors:** Kilian Kluge, Holger Einspieler, David Haberl, Clemens Spielvogel, Dominik Amereller, Gerda Egger, Gero Kramer, Bernhard Grubmüller, Shahrokh Shariat, Marcus Hacker, Lukas Kenner, Alexander Haug

**Affiliations:** 1 Department of Biomedical Imaging and Image-Guided Therapy, Division of Nuclear Medicine, Medical University of Vienna, Währinger Gürtel 18-20, 1090 Vienna, Austria; 2 Christian Doppler Laboratory for Applied Metabolomics (CDL AM), Medical University of Vienna, Vienna, Austria; 3 Department of Pathology, Medical University of Vienna, Vienna, Austria; 4 Department of Urology, Medical University of Vienna, Vienna, Austria; 5 Department of Urology and Andrology, University Hospital Krems, Krems, Austria; 6 Karl Landsteiner University of Health Sciences, Krems, Austria; 7 Karl Landsteiner Institute of Urology and Andrology, Vienna, Austria; 8 Department of Urology, University of Texas Southwestern Medical Center, Dallas, TX USA; 9 Department of Special Surgery, Division of Urology, The University of Jordan, Amman, Jordan; 10 Department of Urology, Second Faculty of Medicine, Charles University, Prague, Czech Republic; 11 Department of Urology, Weill Cornell Medical College, New York, NY USA

**Keywords:** Liquid biopsy, ctDNA, Prostate cancer, PSMA, PET/CT

## Abstract

**Background:**

Circulating-tumor DNA (ctDNA) and prostate-specific membrane antigen (PSMA) ligand positron-emission tomography (PET) enable minimal-invasive prostate cancer (PCa) detection and survival prognostication. The present study aims to compare their tumor discovery abilities and prognostic values.

**Methods:**

One hundred thirty men with confirmed PCa (70.5 ± 8.0 years) who underwent [^68^Ga]Ga-PSMA-11 PET/CT (184.8 ± 19.7 MBq) imaging and plasma sample collection (March 2019–August 2021) were included. Plasma-extracted cell-free DNA was subjected to whole-genome-based ctDNA analysis. PSMA-positive tumor lesions were delineated and their quantitative parameters extracted. ctDNA and PSMA PET/CT discovery rates were compared, and the prognostic value for overall survival (OS) was evaluated.

**Results:**

PSMA PET discovery rates according to castration status and PSA ranges did differ significantly (*P* = 0.013,* P* < 0.001*)*, while ctDNA discovery rates did not (*P* = 0.311, *P* = 0.123). ctDNA discovery rates differed between localized and metastatic disease (*P* = 0.013). Correlations between ctDNA concentrations and PSMA-positive tumor volume (PSMA-TV) were significant in all (*r* = 0.42, *P* < 0.001) and castration-resistant (*r* = 0.65, *P* < 0.001), however not in hormone-sensitive patients (*r* = 0.15, *P* = 0.249). PSMA-TV and ctDNA levels were associated with survival outcomes in the Logrank (*P* < 0.0001, *P* < 0.0001) and multivariate Cox regression analysis (*P* = 0.0023, *P* < 0.0001).

**Conclusion:**

These findings suggest that PSMA PET imaging outperforms ctDNA analysis in detecting prostate cancer across the whole spectrum of disease, while both modalities are independently highly prognostic for survival outcomes.

**Graphical Abstract:**

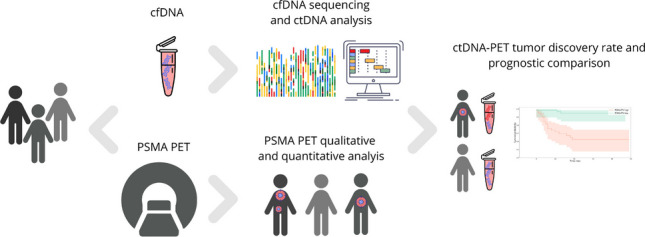

**Supplementary Information:**

The online version contains supplementary material available at 10.1007/s00259-024-06698-7.

## Introduction

Prostate cancer (PCa) remains a leading cause of mortality among men [1] despite recent diagnostic and therapeutic advancements [2].

Clinically, PCas exhibit significant heterogeneity, with overall survival (OS) rates ranging from nearly 100% over 5 years for early, localized, and hormone-sensitive PCa (hsPC) to mere months in advanced castration-resistant (CRPC) disease [3]. This variability is partly due to changes in disease extent and the underlying tumor genotype [4], which influences treatment options and therapeutic resistances. This makes periodic reevaluation of tumor progression and patient prognosis crucial for effective and timely clinical decision-making [5].

Molecular imaging using prostate-specific membrane antigen [6] (PSMA) ligand positron-emission tomography (PET) has transformed PCa imaging, outperforming conventional imaging modalities [7, 8], particularly in detecting local relapses and metastasis at low PSA levels [9]. Its semi-quantitative analysis also yields prognostic outcome information, especially for advanced disease stages [10–12].

Similarly, analyzing plasma-derived cell-free DNA (cfDNA) [13], particularly its circulating-tumor DNA (ctDNA) fraction (ctDNA%) through next-generation sequencing (NGS), has proven valuable in identifying biomarkers for survival [14, 15] and treatment responses [16, 17]. While deep sequencing offers detailed genomic insights [18, 19], its high costs have led to the adoption of low-pass whole-genome sequencing (lpWGS) [14, 15, 20] as a cost-effective alternative. lpWGS sequencing allows for the detection of copy number variation (CNV), a genomic hallmark of PCa [21], and thereby ctDNA quantification, which can enable tumor detection [22, 23] and is known to be prognostic of PCa survival outcomes [14, 15].

As the landscape of minimal-invasive methodologies for tumor detection and prognosis evolves, biomarker comparisons become essential to inform clinicians about the optimal applications of each methodology and clinical scientists about the most promising applications in future studies.

To date, such comparative radiogenomic studies have, inter alia, focused on the relationship between genomic aberrations and multiparametric magnetic resonance imaging (mpMRI) findings in suspected PCa [24] and spatial interlesional PSMA PET heterogeneity [25] in advanced disease.

However, the comparative diagnostic and prognostic utility of ctDNA levels and PSMA PET-based estimates of tumor burden has not been elucidated.

We hypothesized that [^68^Ga]Ga-PSMA-11 PET/CT provides greater sensitivity in detecting PCa compared to ctDNA; however, that both methods would yield associated measures of tumor burden and prognosis.

## Methods

### Study design

In this retrospective single-center study with prospective sample collection conducted at the Medical University of Vienna (March 2019–August 2021), 187 men with confirmed PCa referred for [^68^Ga]Ga-PSMA-11 PET/CT underwent PET/CT imaging and blood sample collection. An all-comer recruitment strategy was employed. All patients gave their written informed consent for imaging, blood sample collection, and associated analysis. This study was approved by the ethics committee of the Medical University of Vienna (ID: 1649/2016).

**Fig. 1:**
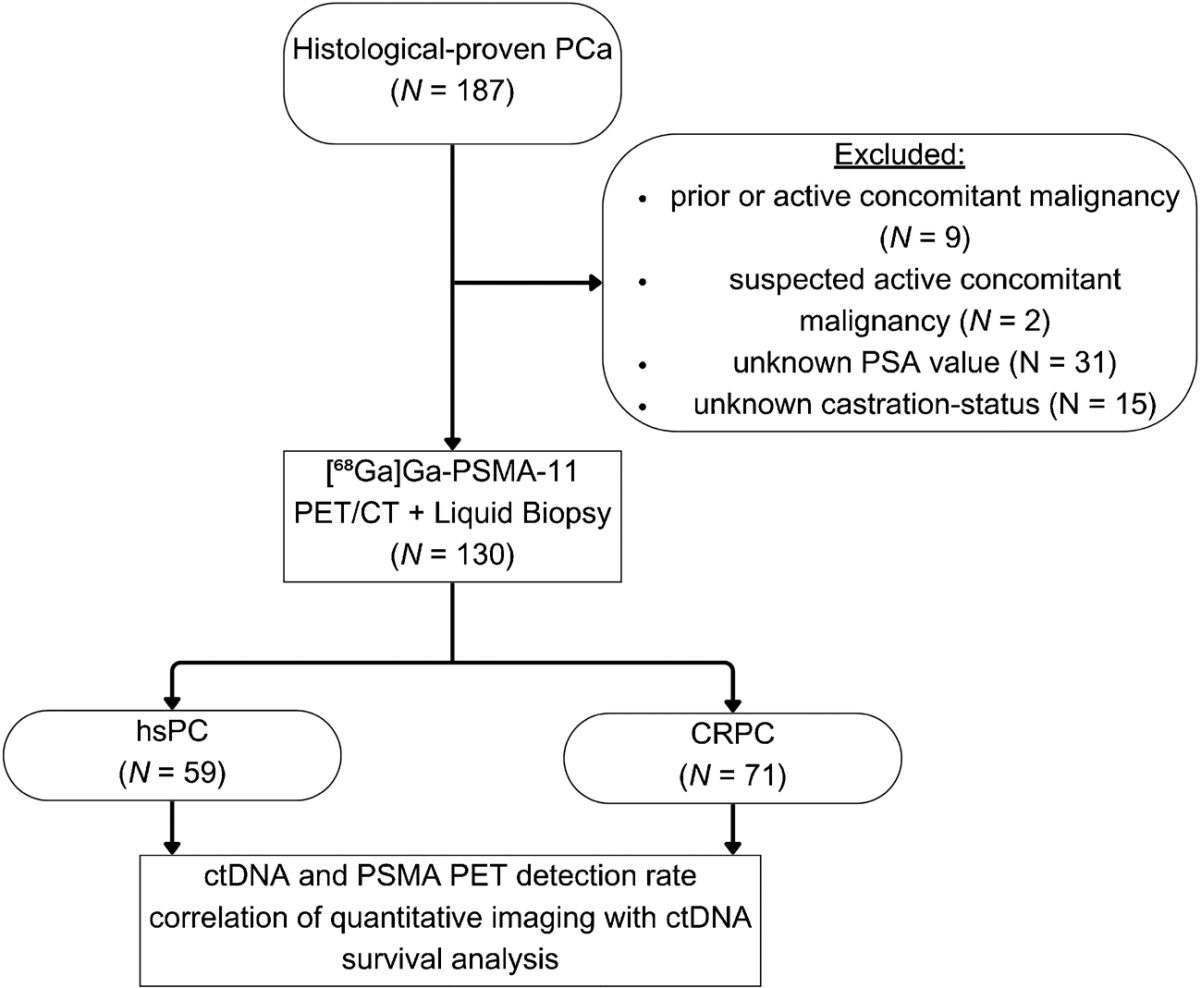
Consort diagram

For this analysis, patients with histologically proven PCa, known PSA levels, and castration status were included, while patients with active or a history of concomitant malignancies other than PCa (*N* = 11), unknown PSA values (*N* = 31), and unknown castration status (*N* = 15) were excluded (Fig. 1).

Clinical data, such as PSA levels, castration status, and pre-, concurrent, and post-imaging therapy data, were gleaned retrospectively from the medical records. Follow-up and overall survival (OS) data (censorization 13th August 2023) were sourced from the National Health Statistical Service. The primary endpoints of this study were (a) ctDNA and PSMA PET/CT tumor signal discovery rates according to castration status and PSA levels, (b) the relationship of ctDNA concentrations and the PSMA-TV in all patients and according to their respective castration status, and (c) the prognostic value of ctDNA and PSMA-TV levels with regard to overall survival (OS).

### Plasma sample collection, cfDNA extraction, quantification, and sequencing

Before tracer injection, blood samples were collected in Cell-Free DNA BCT tubes (Streck Inc., Nebraska, USA). cfDNA was extracted from the plasma using the QIAamp Circulating Nucleic Acid Kit (QIAGEN, Venlo, Netherlands), following the manufacturer’s procedure. cfDNA sample quantities were assessed using the Fragment Analyzer system and the HS NGS Fragment Kit (respectively Agilent, California, USA), as per the manufacturer’s guidelines. Next, Fragment Analyzer results were analyzed with the PROSize software (v2.0, Agilent, California, USA) for automatic DNA concentration calculation. cfDNA quantities are expressed as ng/µL.

DNA sequencing libraries were prepared from 19.5 µl of isolated DNA using the xGEN EZ UNI Library preparation kit combined with stubby adaptors (IDT, Iowa, USA, respectively) containing 3 bp random sequence used as UMI. Library PCR amplification was carried out with the xGEN EZ UNI Library preparation kit in combination with KAPA UDI primers (Roche, Switzerland). Samples were sequenced on NovaSeq 6000 (Illumina, California, USA) in a paired-end 2 × 60 bp setting.

### Bioinformatic analysis

We developed an in-house method to analyze the ctDNA fraction in blood samples using low-coverage WGS sequencing. Raw sequencing reads were initially mapped to the human genomic reference GRCh38 using the BWA tool [26]. Next, mapped raw sequencing reads were counted in 500 kb bin intervals. These bin counts underwent normalization based on sample size and GC content to address biases and variations. From this data, we determined an initial, approximate ctDNA fraction using the density plots of the bin sizes. To call CNVs, we employed an algorithm using the normalized binned read counts, incorporating a negative binomial distribution for individual bin counts to handle overdispersion similar to the ichorCNA methodology [20]. This was followed by using a dynamic Bayesian network model for holistic CNV predictions. The procedure was iterative, with CNVs re-called based on the updated ctDNA fraction and the ctDNA fractions recalculated using the new CNV predictions. In cases where no CNVs were discerned, we assigned the ctDNA fraction a default value of 0.05 and repeated the CNV calling. We measured the quality of our modelled CNVs and ctDNA by examining the residual difference between the actual bin sizes and the sizes predicted post-CNV and ctDNA adjustments. To test the significance of our predicted ctDNA for each sample, we compared residuals from our primary model to those from a noise model created using the same bin count data but with randomly permuted bins, employing the Kolmogorov–Smirnov Test for this purpose. Our final results excluded samples without called CNVs, those with a Kolmogorov–Smirnov Test *P*-value greater than 0.05, and samples predominantly predicting deletions around chromosome centromeres—a potential sign of an unidentified technical bias (Supplementary Material—Methods).

### Imaging protocol and image analysis

Patients were given an intravenous injection of 184.8 MBq (± 19.7 SD) of [^68^Ga]Ga-PSMA-11 and scanned on Biograph TruePoint PET/CT scanner (Siemens Healthineers, Erlangen, Germany) from the skull base to the upper femur an hour post-injection. First, CT scans were taken at 120 kv and 230 mAs and intravenous contrast, except contraindications for contrast applications existed, followed by PET scan acquisition in 3–4 bed positions and iterative reconstruction.

Two nuclear medicine physicians analyzed the images on a dedicated workstation using the Hybrid 3D software (v4.0.0, Hermes Medical Solutions, Stockholm, Sweden), delineating and labelling all PSMA-expressing primary and secondary tumor lesions by their anatomical locations. Lesion identification was performed qualitatively, informed by liver uptake, followed by semiautomatic delineation using a region-growing algorithm (Hybrid 3D software, v4.0.0). The PSMA-TV and standardized uptake values (SUV) normalized to body weight were extracted both from an aggregated master lesion and per anatomic region. The anatomic tumor lesion region that contributed most to the overall PSMA-TV was defined as the dominant tumor fraction (Supplementary Material—Methods).

### Statistical analysis

Continuous variables are reported as mean (± SD), and categorical outcomes as frequencies (%). ctDNA and PSMA PET tumor signal discovery rates and dominant fraction’s association with castration status, PSA ranges, and disease extent were evaluated using Chi-squared and Fisher’s tests. Non-normalized and PSMA-TV-normalized ctDNA concentrations were compared using the Kruskal–Wallis test after assessing normality and heteroskedasticity with Shapiro–Wilk and Levene’s tests. Post-hoc analysis was conducted using Dunn-Bonferoni’s test if the null hypothesis was rejected. The correlation between ctDNA concentrations and PSMA-TV was determined using Spearman’s coefficient. PSMA-TV’s predictive value for ctDNA discovery was analyzed using the area under the receiver-operating-characteristic curves (AUC). OS probabilities were estimated using Kaplan–Meier estimates, and differences in survival distributions were evaluated with the non-parametric Logrank test. The relationship between OS and ctDNA concentration and PSMA-TV was examined using multivariate Cox regression analysis after checking data for multicollinearity and proportional hazard with Belsley-Kuh-Welsch and Schoenfeld residuals. All analyses assumed a 5% alpha risk. All confidence intervals (CI) are 95% CIs. Statistical analyses were conducted using EasyMedStat software (v3.24, EasyMedStat, Paris, France) (Supplementary Material—Methods). For details on the exploratory machine learning analysis of imaging- and plasma-derived features in their predictive ability for OS in single and combined use, see Supplementary Material—Methods.

## Results

### Clinical cohort

In total, 130 men with confirmed PCa (age 70.5 ± 8.0 years, PSA 96.35 ± 438.44) who underwent [^68^Ga]Ga-PSMA-11 PET/CT imaging and plasma sample collection were analyzed. The demographic and clinical characteristics are presented in Table 1.

**Table 1:** Demographic and clinical patient data

Variable	Total *N* = 130	hsPC *N* = 59	CRPC *N* = 71
Age at inclusion [y]	70.52 (± 7.95) Range: (49.0; 85.0)	69.19 (± 8.3) Range: (50.0; 85.0)	71.63 (± 7.52) Range: (49.0; 85.0)
Tracer dose [MBq]	184.82 (± 19.67) Range: (134.0; 300.0)	185.68 (± 22.14) Range: (134.0; 300.0)	184.11 (± 17.53) Range: (149.0; 263.0)
ctDNA detected			
Yes	40 (30.77%)	15 (25.42%)	25 (35.21%)
No	90 (69.23%)	44 (74.58%)	46 (64.79%)
ctDNA [ng/µL]	0.09 (± 0.41) Range: (0.0; 3.12)	0.00685 (± 0.0178) Range: (0.0; 0.118)	0.156 (± 0.541) Range: (0.0; 3.12)
PSA [ng/ml]	96.35 (± 438.44) Range: (0.01; 3689.0)	12.45 (± 42.31) Range: (0.09; 317.0)	166.06 (± 584.73) Range: (0.01; 3689.0)
PSMA-TV [cm^3^]	109.74 (± 294.64) Range: (0.0; 1597.67)	15.49 (± 85.81) Range: (0.0; 659.07)	188.07 (± 374.4) Range: (0.0; 1597.67)
PSMA positive lesion			
Any lesion	102 (78.46%)	40 (67.8%)	62 (87.32%)
Prostate lesion	42 (32.31%)	21 (35.59%)	21 (29.58%)
Lymph node lesion	53 (40.77%)	20 (33.9%)	33 (46.48%)
Bone lesion	54 (41.54%)	9 (15.25%)	45 (63.38%)
Organ lesion	18 (13.85%)	4 (6.78%)	14 (19.72%)
Dominant fraction			
Prostate	25 (19.23%)	17 (28.81%)	8 (11.27%)
LN	31 (23.85%)	16 (27.12%)	15 (21.13%)
Bone	44 (33.85%)	6 (10.17%)	38 (53.52%)
Organ	3 (2.31%)	2 (3.39%)	1 (1.41%)
Systemic therapies while PET			
Antihormonal therapies	55 (42.31%)	1 (1.69%)	54 (76.06%)
Cytotoxic therapies	4 (3.08%)	0 (0.0%)	4 (5.63%)
Systemic therapies after PET			
Local	31 (39.24%)	22 (61.11%)	9 (20.93%)
Local + ADT	5 (6.33%)	3 (8.33%)	2 (4.65%)
ADT	20 (25.32%)	8 (22.22%)	12 (27.91%)
CHT	2 (2.53%)	0 (0.0%)	2 (4.65%)
CHT + ADT	2 (2.53%)	1 (2.78%)	1 (2.33%)
177Lu-PSMA	17 (21.52%)	1 (2.78%)	16 (37.21%)
Study	2 (2.53%)	1 (2.78%)	1 (2.33%)
Mean follow-up [m]	19.23 (± 13.56) Range: (0.2; 49.5)	21.25 (± 13.37) Range: (0.2; 47.9)	17.55 (± 13.57) Range: (0.4; 49.5)

Qualitative data as numbers and percentages; continuous data as mean, standard deviation and range; Local disease comprised of prostate and seminal vesicle lesions

*ADT* androgen-deprivation therapy, *CHT* chemotherapy, *ctDNA* circulating-tumor DNA

### PSMA PET and ctDNA discovery rates per PSA levels and castration status

**Fig. 2:**
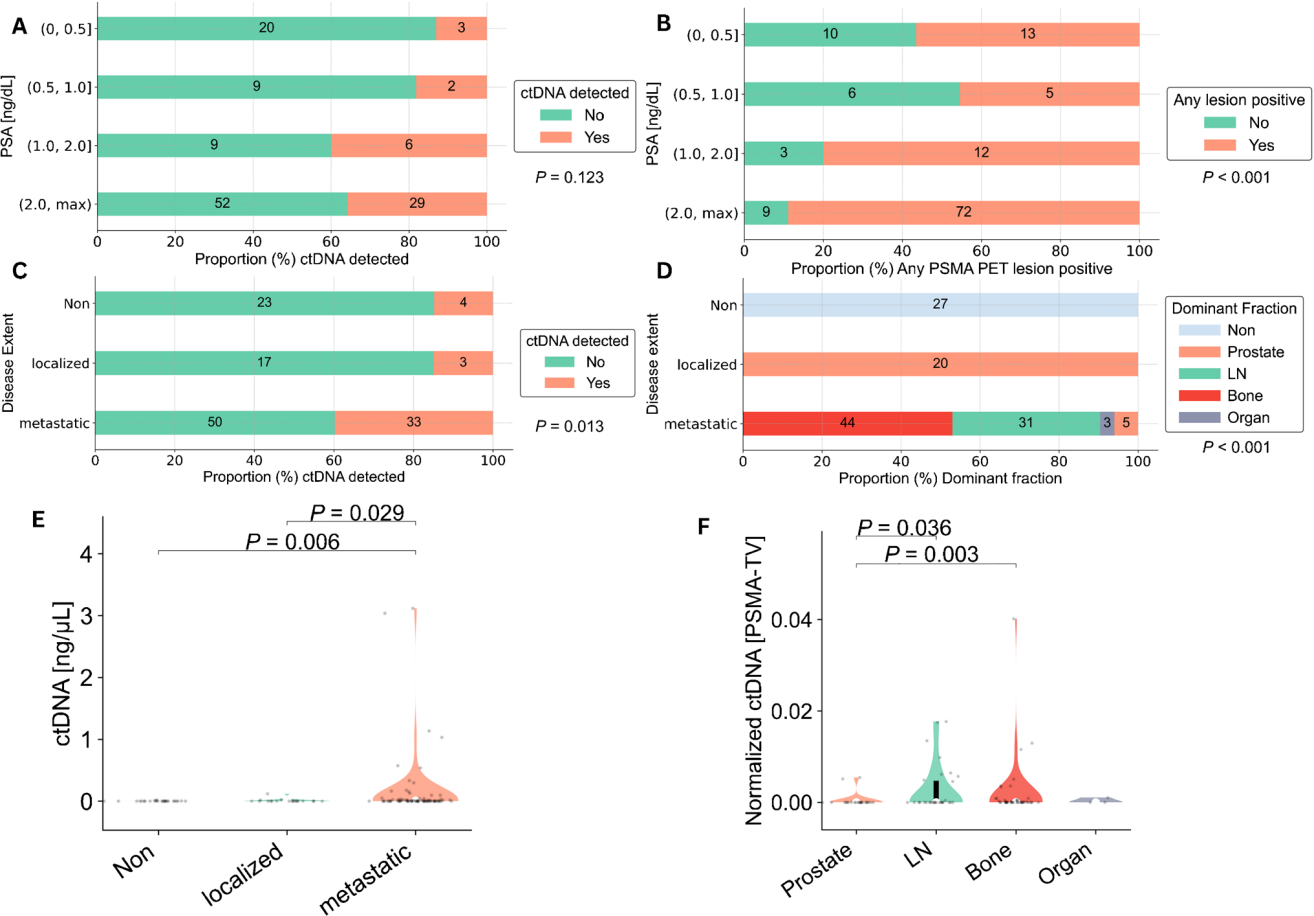
ctDNA and PSMA PET discovery rates as well as their findings per disease extent. ctDNA and PSMA PET discovery rates across different PSA ranges (**A**, **B**). ctDNA discovery rates according to PSMA PET assessed disease extent (**C**). Dominant lesion fraction contributing most to respective PSMA PET lesion extent (**D**). Violin plots depicting ctDNA levels relative to lesion extent (**E**). Violin plots illustrating PSMA-TV normalized ctDNA levels according to the dominant lesion fraction (**F**). ctDNA, circulating-tumor DNA; PSMA-TV, PSMA tumor volume

The PSMA PET and ctDNA analysis detected tumor signals (discovery rates) in 56.52% and 13.04%, 45.45% and 18.18%, 80.0% and 40.0%, and 88.89% and 35.8% of patients at the PSA ranges (0, 0.5), (0.5, 1.0), (1.0, 2.0), and (2.0, 3689.0) ng/mL, respectively (*P* < 0.001 and *P* = 0.123, respectively) (Table 2, Fig. 2A, B).

**Table 2:** PSMA PET and ctDNA discovery rate contingency table according to PSA ranges

PSA range	PET positive	PET negative	ctDNA positive	ctDNA negative	Total observations
(0, 0.5]	13 (56.52%)	10 (43.48%)	3 (13.04%)	20 (86.96%)	23 (17.69%)
(0.5, 1.0]	5 (45.45%)	6 (54.55%)	2 (18.18%)	9 (81.82%)	11 (8.46%)
(1.0, 2.0]	12 (80.0%)	3 (20.0%)	6 (40.0%)	9 (60.0%)	15 (11.54%)
(2.0, 3689.0]	72 (88.89%)	9 (11.11%%)	29 (35.8%)	52 (64.2%)	81 (62.31%)
Total	102 (78.46%)	28 (21.54%)	40 (30.77%)	90 (69.23%)	130 (100%)

Data are displayed as *N* and percentages

*ctDNA* circulating tumor DNA, *PSA* prostate-specific antigen, *PET* positron-emission-tomography

The PSMA PET discovery rates between hsPC (67.8%) and CRPC (87.32%) did differ significantly (odds ratio (OR) = 0.31; 95% CI = [0.13, 0.74]; *P* = 0.013), while the ctDNA discovery rates between castration statuses (hsPC, 25.42%; CRPC, 35.21%) did not (*P* = 0.311) (Supplementary Fig. 1A-B).

### Comparison of ctDNA findings with imaged disease extent and dominant lesion fraction

The ctDNA discovery rates were 14.81%, 15.00%, and 39.76% in patients with no lesions, localized, and metastatic disease on PSMA PET imaging, respectively (*P* = 0.013) (Fig. 2C).

Respective median levels of ctDNA according to PSMA PET imaged disease extent differed significantly (*P* = 0.006) and are displayed in Fig. 2E. Post-hoc adjusted, pairwise analyses revealed differences for the metastatic versus no lesion (*P* = 0.006, mean difference CI = [− 0.37, 0.1]) and metastatic versus localized groups (*P* = 0.029, CI = [− 0.4, 0.15]).

The dominant lesion fraction in patients with metastatic disease on imaging was in 6.02% prostate, 37.35% lymph node, 53.01% bone, and 3.61% organ (Fig. 2D). Respective median PSMA-TV-normalized ctDNA levels (normctDNA) according to the dominant fraction of PSMA PET differed significantly (*P* = 0.036) and are shown in Fig. 2F. Pairwise post-hoc analyses revealed only normctDNA differences for the prostate versus bone (*P* = 0.003, CI = [− 0.0021, 0.0051]) and prostate versus lymph node groups (*P* = 0.036, CI = [− 0.0006, 0.0053]) (Fig. 2F).

### Correlation of ctDNA levels with PSMA-TV and determination of ctDNA PSMA-TV discovery threshold

**Fig. 3:**
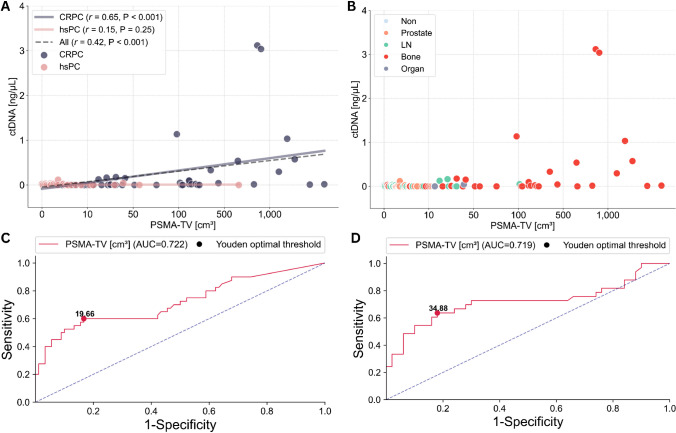
ctDNA and PSMA-TV relationships. Scatter plot displaying the correlation between ctDNA and PSMA-TV levels according to castration status (**A**) and dominant lesion fraction (**B**); x-scales are log-transformed for better scale comparability. ROC curves highlighting the optimal PSMA-TV threshold for ctDNA detection in all (**C**) and patients with metastatic disease on imaging (**D**)

Significant and positive correlations were found between ctDNA levels and the PSMA-TV in all (*r* = 0.42, *P* < 0.001) and CRPC patients (*r* = 0.65, *P* < 0.001), while no significant correlation was observed in hsPC patients (*r* = 0.15, *P* = 0.249) (Fig. 3A, B).

In the overall cohort, the optimal threshold for PSMA-TV to predict ctDNA discovery was 19.66 [cm^3^] according to Youden [27] with an area under the curve (AUC) of the receiver operator curve (ROC) of 0.722 (CI = [0.618, 0.826]), while in metastatic patients, the optimal threshold was 34.88 [cm^3^] with an AUC of 0.719 (CI = [0.592, 0.846]) (Fig. 3C, D).

### Overall survival analysis

**Fig. 4:**
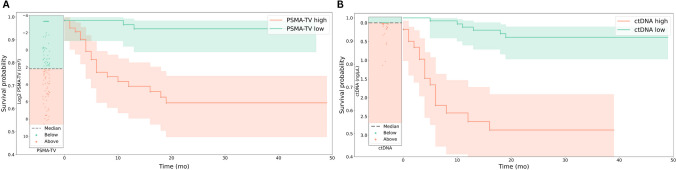
Kaplan–Meier curves representing the survival probabilities between the high and low PSMA-TV (**A**) and high and low ctDNA groups (**B**)

There were significant differences between survival distributions of the PSMA-TV and ctDNA high and low groups (*P* < 0.0001 and *P* < 0.0001, respectively) (Fig. 4A, B).

In the multivariate Cox regression analysis, there were significant hazard differences between the PSMA-TV (hazard ratio (HR) = 6.65, CI = [1.96, 22.52], *P* = 0.0023) and ctDNA high and low groups (HR 7.56, CI = [2.94, 19.42], *P* < 0.0001*)* (Table 3)*.* For the results of the exploratory machine learning analysis of imaging- and plasma-derived features in their predictive ability for OS in single and combined use, see Supplemental Material—Results.

**Table 3:** Multivariate Cox regression of the binary explanatory variables ctDNA and PSMA-TV

Predicted endpoint	Category	Hazard ratio [CI]	*P*-value
Overall survival	**ctDNA group**		** < 0.0001**
	ctDNA low	0.132 [0.0515, 0.34]	
	ctDNA high	7.56 [2.94, 19.42]	
	**PSMA-TV group**		**0.0023**
	PSMA-TV low	0.15 [0.0444, 0.509]	
	PSMA-TV high	6.65 [1.96, 22.52]	

*ctDNA* circulating-tumor DNA, *PSMA-TV* PSMA tumor volume, *CI* confidence interval

## Discussion

Over the last decade, PSMA-ligand PET imaging has redefined benchmarks in PCa imaging [7, 8], excelling in detecting locally relapsed and metastatic disease at very low PSA levels [9] while also providing outcome-relevant semi-quantitative data [10–12]. Simultaneously, the prognostic value of plasma-derived ctDNA has been repeatedly demonstrated in multicentric retrospective [14] and prospective studies [15] alike. However, limitations in ctDNA detection in low-burden PCa have been reported and imaging-based quantitative thresholds remain to be defined [22, 23]. As minimal-invasive tumor detection and outcome prognostication methods continue to evolve, comparative studies become imperative to understand each approach’s relative merits and limitations to inform clinicians about the optimal applications of each methodology and clinical scientists about the most promising applications in future studies.

We, therefore, aimed to elucidate the comparative detection and prognostic efficacy of ctDNA levels and PSMA PET-based estimates of PCa tumor burden.

Consistent with numerous previous studies [9], our findings revealed high sensitivity of PSMA PET in identifying disease even at minimal PSA levels, ranging from 56% PET-positivity at PSA levels below 0.5 ng/mL to roughly 89% at PSA levels exceeding 2 ng/mL (Fig. 2B).

In contrast, merely low ctDNA discovery rates with a non-significant trend towards higher ctDNA detection at higher PSA levels were tangible (Fig. 2A). This is in line with previous studies [22, 23], which reported low ctDNA detection rates at low neoplastic loads. Schweizer et al. [23] previously identified high disease burden, PSA levels exceeding 10 ng/mL, and CRPC as key factors for successful ctDNA detection in a cohort of 93 men with PCa, using a multi-gene NGS panel approach to detect somatic cfDNA alterations. Similarly, Hennigan et al. sought to elucidate whether ctDNA was detectable in 112 patients with localized PCa prior to radical prostatectomy and 7 patients with metastatic PCa by ultra-lpWGS and tumor-informed focused resequencing and reported that ctDNA was only detectable in metastatic samples, generally corroborating our findings of significant differences in ctDNA detection between localized and metastatic patients (Fig. 2C, D).

Along these lines, we observed significant positive correlations between ctDNA concentrations and PSMA-TV in the overall cohort and the CRPC groups, while no such correlation was found or trending in the hsPC patients (Fig. 3A). We hypothesize that this association was primarily driven by high-voluminous osseous metastasis present in CRPC patients, which was largely absent in the hsPC cohort (Fig. 3B). Nevertheless, other potentially influencing factors such as tumor biologic-specific shedding dynamics and clearance rates might also influence correlations between ctDNA levels and tumor burden, particularly in low-volume cases, which might not be definitively characterized by our small-size mono-centric cohort.

To further examine whether ctDNA shedding—primarily resulting from tumor cell apoptosis and necrosis [28]—varies by tumor organ site, we normalized ctDNA levels (normctDNA) against total PSMA-TV. We then compared the normctDNA levels according to the underlying dominant lesion fraction—that being the anatomic region contributing most to the overall tumor volume. In line with Hennigan et al. [22], our analysis revealed significantly higher ctDNA levels between lymph node and bone metastasis relative to prostate lesions, though no differences were noted between lymph node and bone metastasis themselves (Fig. 2F). This suggests that lymph node and bone lesions do not differ in ctDNA shedding rates, corroborating the notion that variations in ctDNA levels are primarily driven by overall tumor burden (Figs. 2E, 3A), which was highest in predominantly osseously metastasized patients (Fig. 3B).

Next, we sought to establish optimal volume thresholds for the successful lpWGS-based ctDNA detection by ROC analyses (Fig. 3C, D), to facilitate potential future study designs which aim to incorporate liquid biopsy approaches.

In summary, our data suggests that PSMA PET imaging strongly surpassed ctDNA in its discovery efficacy for PCa [9] across the whole spectrum of disease, in line with previous reports of PSMA PETs’ high sensitivity in detecting tumor lesions and low abundance of ctDNA in low-volume [23] and non-metastatic prostate cancer [22], indicating that ctDNA analysis might be most applicable in the setting of metastatic CRPC in the future.

As the individual prognostic utilities of ctDNA [14, 15] and PSMA-PET-derived estimates of tumor burden [29] are well established, we sought to explore their relative comparative value for survival prediction. We, therefore, conducted a Logrank survival analysis using the median values of ctDNA and PSMA-TV levels as a cutoff and observed significantly different survival distributions between the high and low-level ctDNA and PSMA-TV groups (Fig. 4A, B), which is in line with previous reports [10–12, 14, 15]. A multivariate regression analysis affirmed their independent association with overall survival, which yielded comparable HR for the ctDNA and PSMA-TV stratifiers (Table 3).

In summary, both ctDNA and PSMA-PET imaging appear to provide strong and independent predictive values for survival outcomes. However, the study’s relatively brief follow-up period might not fully represent changes over time, warranting cautious interpretation of their prognostic significance.

While our study provided insights into the comparative discovery rates and prognostic utility of ctDNA and PSMA PET in prostate cancer, its limitations need to be acknowledged.

First of all, as a small retrospective study with prospective sample collection, we are prone to recall bias and selection bias, which we tried to mitigate by excluding patients with inconclusive records and by employing an all-comer recruitment strategy. Next, due to the cross-sectional design including patients with disseminated and advanced disease, no histological ground truth was included allowing only for a descriptive comparison of the two methods as well as to previously published work.

Another potential limitation of ctDNA detection through CNV analysis with lpWGS at low ctDNA fractions may be an increased likelihood of detecting inherent noise as a CNV signal, potentially yielding false positive results. However, we tried to mitigate this by excluding samples with non-significant CNV profiles compared to a noise model and CNV profiles indicative of unidentified technical biases. Furthermore, the general accordance of ctDNA detection limits with prior work [22, 23] and demonstrated predictive potential supports its biological validity.

Last, while our findings are generally in line with previous reports of PSMA PETs and ctDNA discovery rates and prognostic potential, the direct clinical interpretability might be partially limited due to the biological and therapeutic heterogeneity of the studied cohort, which did not allow for more in-depth assessment of the influence of potential confounders.

Despite its constraints, it is crucial to stress is merits. Blood sampling prior to tracer injection allowed for a synchronous comparative perspective of ctDNA levels and PSMA PET findings, while the inclusion of roughly equal parts of hsPC and CRPC allowed for an exploration of the best clinical scenarios to use ctDNA in future studies.

As ctDNA levels appear to be present in sufficient quantities for analysis in metastatic disease and the need for outcome risk stratifying and therapy predicting biomarkers is high in advanced CRPC, future studies should explore if the incorporation of ctDNA analysis into currently insufficient imaging-based selection strategies for PSMA-radioligand therapies allows for enhanced tumor biology profiling and thereby more personalized disease management.

## Conclusion

These findings suggest that PSMA PET imaging outperforms lpWGS-based ctDNA analysis in detecting prostate cancer across the whole spectrum of disease, with both modalities being independently highly prognostic for survival outcomes.

### Supplementary Information

Below is the link to the electronic supplementary material.

Supplementary file1 (PDF 7.57 MB)

## Data Availability

Data is available upon reasonable request from the corresponding author.
