# Supplementary material for: Comparison of discovery rates and prognostic utility of [68Ga]Ga-PSMA-11 PET/CT and circulating tumor DNA in prostate cancer—a cross-sectional study
Source: Eur J Nucl Med Mol Imaging. 2024 May 2;51(9):2833–42. doi: 10.1007/s00259-024-06698-7 (PMC11224100; doi:10.1007/s00259-024-06698-7)
Supplement: Supplementary file 1 — Supplementary file1 (PDF 7.57 MB) [file 259_2024_6698_MOESM1_ESM.pdf]

Supplemental Material & Data

Comparison of Discovery Rates and Prognostic Utility of [^{68}Ga]Ga-PSMA-11 PET/CT and Circulating Tumor DNA in Prostate Cancer - a Cross-Sectional Study

Kilian Kluge[1,2*], Holger Einspieler[1], David Haberl[1,2], Clemens Spielvogel[1,2], Dominik Amereller[1], Gerda Egger[3], Gero Kramer[4], Bernhard Grubmüller[5-6], Shahrokh Shariat[4,7-11], Marcus Hacker[1], Lukas Kenner[2,3], Alexander Haug[1,2#]

1 Department of Biomedical Imaging and Image-Guided Therapy, Division of Nuclear Medicine, Medical University of Vienna, Vienna, Austria
2 Christian Doppler Laboratory for Applied Metabolomics (CDL AM), Medical University of Vienna, Vienna, Austria
3 Department of Pathology, Medical University of Vienna, Vienna, Austria
4 Department of Urology, Medical University of Vienna, Austria
5 Department of Urology and Andrology, University Hospital Krems, Krems, Austria
6 Karl Landsteiner University of Health Sciences, Krems, Austria
7 Karl Landsteiner Institute of Urology and Andrology, Vienna, Austria
8 Department of Urology, University of Texas Southwestern Medical Center, Dallas, USA
9 Division of Urology, Department of Special Surgery, The University of Jordan, Amman, Jordan
10 Department of Urology, Second Faculty of Medicine, Charles University, Prague, Czech Republic
11 Department of Urology, Weill Cornell Medical College, New York, USA

*First Author
Kilian Kluge, Division of Nuclear Medicine, Medical University of Vienna, Währinger Gürtel 18-20, 1090 Vienna, Austria
kilian.kluge@meduniwien.ac.at
Nuclear Medicine Resident Physician
+43 (0)1 40400 - 58739
ORCID: 0000-0002-4224-7314

#Correspondence:
Alexander Haug, Division of Nuclear Medicine, Medical University of Vienna, Währinger Gürtel 18-20, 1090 Vienna, Austria,
alexander.haug@meduniwien.ac.at
+43 (0)1 40400 - 58744

Table of Contents

Table of Contents
Methods
- Study design
- Plasma sample collection and storage
- Imaging protocol
- Image analysis
- DNA extraction, quantification
- DNA sequencing
- Bioinformatic analysis
- Statistical analysis
- Machine learning workflow

Results
- ctDNA and PSMA PET discovery rate differences according to castration status
- Clinical and demographic table of patients used in 1-year OS prediction
- Compound model performances and feature importances
- Imaging model performances and feature importances
- Plasma model performance and feature importance
- Comparative overview of best-performing machine learning classifiers

Appendix
References
Example sequencing reports and statistics

Methods

Study design

In this retrospective single-centre study with prospective sample collection conducted at the Medical University of Vienna (March 2019 - August 2021), 187 men with confirmed PCa referred for [^{68}Ga]Ga-PSMA-11 PET/CT underwent PET/CT imaging and blood sample collection. An all-comer recruitment strategy was employed. All patients gave their written informed consent for imaging, blood sample collection and associated analysis. This study was approved by the ethics committee of the Medical University of Vienna (ID: 1649/2016). For this analysis, patients with histologically proven PCA, known PSA levels and castration status were included, while patients with active or a history of concomitant malignancies other than PCA (N = 11), unknown PSA values (N = 31) and unknown castration status (N = 15) were excluded. (Figure 1)

Clinical data, such as PSA levels, castration status and pre-, concurrent and post-imaging therapy data, were gleaned retrospectively from the medical records. hsPC was defined as PCa, which was not subjected to prior antiandrogens or did not advance in an castration environment. CRPC as PCa which progressed despite antiandrogen treatment in a castration environment [1].

Follow-up and overall survival (OS) data (censorization 13th August 2023) were sourced from Statistic Austria, the national health statistical service. The primary endpoints of this study were a) ctDNA and PSMA PET/CT discovery rates according to PSA levels, b) the relationship of ctDNA concentrations and the PSMA-TV in all patients and according to their respective castration status and c) the prognostic value of ctDNA and PSMA-TV levels with regard to overall survival (OS).

Plasma sample collection and storage

Prior to tracer injection, blood samples were collected in Cell-Free DNA BCT tubes (Streck Inc., Nebraska, USA). Then, the collected samples were centrifuged twice (1. round: 2000g for 20 min., 2. round: 3200 g for 30 min.) to completely remove of any cellular debris. Afterwards, the derived plasma was stored at –80 °C till further processing.

Imaging protocol

Scans were obtained on a Biograph TruePoint PET/CT scanner (Siemens Healthineers, Erlangen, Germany). The patients received an intravenous injection averaging 184.8 MBq (± 19.7 SD) of [^{68}Ga]Ga-PSMA-11. Static whole-body scans were performed from the skull base to the upper thigh one hour after the tracer injection. CT scans were acquired first at 120 kV and 230 mAs with intravenous contrast (CT matrix size 512x512), except contraindications for contrast application existed. Afterwards, PET scans were acquired in 3-4 bed positions with a matrix size of 168x168, followed by iterative reconstruction using a point-spread-function-based algorithm.

Image analysis

Two nuclear medicine physicians interpreted the images on a dedicated workstation using the Hybrid 3D software (version 4.0.0, Hermes Medical Solutions, Stockholm, Sweden). All primary and secondary PSMA-expressing tumour lesions from the skull to the upper femur were manually delineated and labelled per their anatomical location (prostate, lymph nodes, bone, organ). Lesion identification was performed qualitatively, informed by liver uptake, followed by semiautomatic delineation using a region-growing algorithm (Hybrid 3D software, version 4.0.0). The PSMA-TV, standardized uptake values (SUV) were extracted from an aggregated master lesion, comprised of all delineated lesions, as well as per anatomical region. The anatomic tumor region which contributed most to the overall PSMA-TV was defined as the dominant tumor fraction.

DNA extraction, quantification

The cfDNA was extracted from the stored plasma using the QIAamp Circulating Nucleic Acid Kit (QIAGEN, Venlo, Netherlands) according to the manufacturer's instructions from 4 mL of plasma. Subsequently, the extracted cfDNA was stored at -20°C until further analysis.
The cfDNA was quantified on the Fragment Analyzer (Agilent, California, USA) system using the HS NGS Fragment Kit (Agilent, California, USA), according to the manufacturer's instructions. Electropherograms were read and analysed using the PROSize software (version 2.0, Agilent, California, USA), enabling the calculation of areas under the curves proportional to the DNA concentration of each sample, thereby quantifying the cfDNA and proportion of size fractions in the sample. The cfDNA concentrations are expressed as nanograms (ng) per microliter (µL) of elution volume.

DNA sequencing

DNA sequencing libraries were prepared from 19,5 µl of isolated DNA using the xGEN EZ UNI Library preparation kit (IDT, Iowa, USA) combined with stubby adaptors (IDT, Iowa, USA) containing 3bp random sequence used as UMI. Enzymatic fragmentation time was set to 2 minutes. Library PCR amplification was carried out with the xGEN EZ UNI Library preparation kit in combination with KAPA UDI primers (Roche, Switzerland). Samples were sequenced on NovaSeq 6000 (Illumina, California, USA) in a paired-end 2x60bp setting.

Bioinformatic analysis

We developed an in-house method to analyze the ctDNA fraction in blood samples using low-coverage WGS sequencing. Raw sequencing reads were initially mapped to the human genomic reference GRCh38 using the BWA tool [2]. After mapping the raw sequencing reads to a reference genome, they were counted in 500kb bin intervals. These bin counts underwent normalization based on sample size and GC content to address biases and variations. From this data, we determined an initial, approximate ctDNA fraction using the density plots of the bin sizes. To call CNVs, we employed an algorithm using the normalized binned read counts, incorporating a negative binomial distribution for individual bin counts to handle overdispersion similar to the ichorCNA methodology [3]. This was followed by using a dynamic Bayesian network model for holistic CNV predictions. The procedure was iterative, with CNVs re-called based on the updated ctDNA fraction and the ctDNA fractions recalculated using the new CNV predictions. In cases where no CNVs were discerned, we assigned the ctDNA fraction a default value of 0.05 and repeated the CNV calling. We measured the quality of our modelled CNVs and ctDNA by examining the residual difference between the actual bin sizes and the sizes predicted post-CNV and ctDNA adjustments. To test the significance of our predicted ctDNA for each sample, we compared residuals from our primary model to those from a noise model created using the same bin count data but with randomly permuted bins, employing the Kolmogorov-Smirnov Test for this purpose. Our final results excluded samples without called CNVs, those with a Kolmogorov-Smirnov Test p-value greater than 0.05, and samples predominantly predicting deletions around chromosome centromeres — a potential sign of an unidentified technical bias.

Statistical analysis

Continuous variables are expressed as mean (± standard deviation (SD)), while categorical outcomes are expressed as absolute and relative (%) frequencies.

The association of the ctDNA and PSMA PET tumor signal discovery rates and PSMA PET dominant fraction with castration status, PSA ranges, and disease extent were assessed using the Chi-squared and Fisher's tests, based on the underlying contingency table data distribution.

For the comparison of non-normalized and PSMA-TV normalized ctDNA concentrations according to PSMA PET disease extent and dominant lesion fraction respectively, normality and heteroskedasticity were evaluated using the Shapiro-Wilk and Levene's test, respectively, followed by difference testing using the Kruskal-Wallis test. In case the null hypothesis was rejected, post-hoc adjusted pairwise analysis was performed with Dunn-Bonferoni's test.

The normality of ctDNA concentration and PSMA-TV was evaluated with the Shapiro-Wilk test. Spearman's coefficient was used to assess the correlation between ctDNA concentrations and PSMA-TV, judging correlations as very strong from 1 to 0.9, strong from 0.9 to 0.7, moderate from 0.7 to 0.5, low from 0.5 to 0.3 and weak from 0.3 to 0.

Receiver-operating-characteristic curves were used to assess PSMA-TV's ability to predict ctDNA discovery in all and metastatic patients, expressed as area under the curves (AUCs) and 95% confidence intervals (CI).

OS probabilities and their pointwise 95% CI from the date of inclusion till death were estimated using the Kaplan-Meier method. Survival distributions between high and low ctDNA and PSMA-TV groups (cutoff respective median values) as well as between the cross-validated machine learning classified groups were compared using the non-parametric Logrank test.

To evaluate the relationship between OS and the binary explanatory variables ctDNA concentration and PSMA-TV a multivariate Cox regression analysis was performed after checking data for multicollinearity and proportional hazards with the Belsley-Kuh-Welsch technique and Schoenfeld residuals, respectively.

The alpha risk was set for all statistical analyses to 5% ($\alpha = 0.05$). All CIs are 95% CI.

Statistical analysis was performed with the EasyMedStat software (version 3.24, EasyMedStat, Paris, France).

Machine learning workflow

To explore potential non-linear relationships between imaging and plasma-derived variables and 1-year OS, a 100-fold Monte Carlo (MC) cross-validated machine learning scheme was employed. To evaluate the incremental predictive value of combining ctDNA analysis and PSMA PET, a compound modelling scheme, incorporating imaging and plasma-derived markers, as well as an imaging-only and plasma-only modelling scheme were trained and

their performances compared. All features used in the respective ML schemes and their associated definitions are summarized in the supplemental Appendix.

In order to enable robust machine learning performance estimations, 100-fold Monte Carlo (MC) cross-validation schemes using a 80% to 20% training-to-test set ratio were employed. Modelling was strictly conducted on a training data subset. The model performances were exclusively evaluated on the test data.

For each fold, fold-wise preprocessing using feature standardization, k-nearest neighbour feature imputation, minimum-redundancy-maximum-relevance-based (mRMR) [4] feature selection and class balancing using synthetic minority over-sampling technique (SMOTE) [5] was employed. An automated hyperparameter optimization via random search was used. Subsequently, six different machine learning (ML) classifiers were trained on the preprocessed training data, namely decision trees (DT), logistic regression (LGR), k-nearest neighbours (kNN), random forest (RF), extreme gradient boosting (XBG) and explainable boosting machine (EBM) [6]. Model prediction performance was estimated via the MC cross-validation scheme using confusion matrix analytics. True positive, true negative, false positive and false negative confusion matrix entries were calculated by evaluating the validation samples in each fold. Sensitivity (SNS), specificity (SPC), accuracy (ACC), positive predictive values (PPV), negative predictive values (NPV), balanced accuracy (BACC) and area under the receiver operating characteristic (AUC) were used as performance metrics.

In order to explore the relative importance and the directionality of individual features with the predicted outcomes, Shapley additive explanation (SHAP) [7] analysis was employed for each model. SHAP analysis was conducted based on a model developed on the entire dataset. All analyses were conducted using Python (version 3.9.5) using the following packages: Numpy [8], Pandas [9], Seaborn [10], Matplotlib [11], Scikit-learn [12], Imbalanced-learn [13], Shap [14], XGBoost [15], InterpretML [6], mRMR1 [4].

Results

ctDNA and PSMA PET discovery rate differences according to castration status

The PSMA PET (odds ratio (OR) = 0.31; 95% CI=[0.13, 0.74]; *P* = 0.013) and ctDNA discovery rates (OR = 0.63; 95% CI=[0.29, 1.34]; *P* = 0.311) were respectively 67.8% and 25.42% in the hsPC and 87.32% and 35.21% in CRPC patients, respectively (Figure 1A-B).

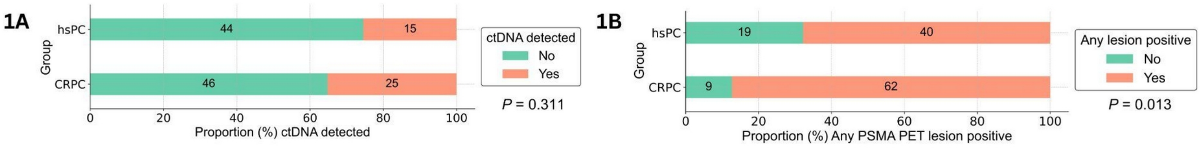

Figure 1: ctDNA and PSMA PET discovery rates according to castration status

Clinical and demographic table of patients used in 1-year OS prediction

A total of 105 patients (age 71.5 ± 7.74 years) were eligible for the 1yOS survival prediction based on the available follow-up and outcome data. Their clinical and demographic characteristics are presented in Table 1. For input features used in machine learning models see supplementary Table 6 (Appendix).

Variable	Prediction cohort<br>N = 105
**Age at inclusion [y]**	71.5 ± 7.74, Range: (49.0 ; 85.0)
**Tracer dose [MBq]**	186.25 (± 20.14), Range: (143.0 ; 300.0)
**ctDNA detected**	
yes	31 (29.52%)
no	74 (70.48%)
**ctDNA [ng/µL]**	0.0897 (± 0.436), Range: (0.0 ; 3.12)
**PSA [ng/mL]**	112.77 (± 486.32), Range: (0.01 ; 3689.0)
**PSMA-TV [cm³]**	114.6 (± 318.59), Range: (0.0 ; 1597.67)
**PSMA positive lesion**	
Any lesion	81 (77.14%)
Prostate lesion	31 (29.52%)
Lymph node lesion	43 (40.95%)
Bone Lesion	43 (40.95%)
Organ Lesion	16 (15.24%)
**Dominant fraction**	
Prostate	19 (18.1%)
LN	16 (27.12%)
Bone	34 (32.38%)
Organ	3 (2.86%)
**Group**	
hsPC	47 (44.76%)
CRPC	58 (55.24%)
**Systemic therapies while PET**	
Antihormonal therapies	44 (41.9%)
Cytotoxic therapies	3 (2.86%)
**Systemic therapies after PET**	
Local	26 (40.62%)
Local + ADT	4 (6.25%)
ADT	16 (25.0%)
CHT	1 (1.56%)
CHT + ADT	2 (3.12%)
177Lu-PSMA	13 (20.31%)
Study	2 (3.12%)
**Mean Follow-up [m]**	23.01 (± 12.25), Range: (0.0 ; 49.0)
**1-year OS**	
yes	85 (80.95%)
no	20 (19.05%)

Table 1. Demographic and clinical patient data of 1yOS survival prediction cohort
Qualitative data as numbers and percentages; Continuous data as mean, standard deviation and range; Local disease comprised of prostate and seminal vesicle lesions

Compound model performances and feature importances

The best-performing compound classifier for the predicted endpoint 1yOS was RF. For the individual performances of all compound classifiers see Table 2. For the most important features according to SHAP analysis of the best-performing model (RF) see Figure 2. For the Kaplan-Meier curves visualizing the survival probability stratification of the best-performing imaging-based classifier see Figure 3.

	ACC [± CI]	SNS [± CI]	SPC [± CI]	PPV [± CI]	NPV [± CI]	BACC [± CI]	AUC [± CI]
**RF**	0.90 [± 0.011]	0.77 [± 0.038]	0.94 [± 0.011]	0.77 [± 0.040]	0.95 [± 0.008]	0.85 [± 0.019]	0.92 [± 0.016]
**kNN**	0.88 [± 0.012]	0.69 [± 0.044]	0.92 [± 0.013]	0.71 [± 0.042]	0.93 [± 0.009]	0.81 [± 0.022]	0.85 [± 0.023]
**XGB**	0.90 [± 0.013]	0.75 [± 0.043]	0.93 [± 0.011]	0.74 [± 0.040]	0.94 [± 0.009]	0.84 [± 0.022]	0.91 [± 0.020]
**EBM**	0.90 [± 0.012]	0.78 [± 0.036]	0.93 [± 0.013]	0.75 [± 0.034]	0.95 [± 0.008]	0.85 [± 0.019]	0.90 [± 0.018]
**SVM**	0.87 [± 0.015]	0.70 [± 0.047]	0.91 [± 0.017]	0.70 [± 0.043]	0.93 [± 0.010]	0.80 [± 0.023]	0.87 [± 0.023]
**LGR**	0.86 [± 0.015]	0.75 [± 0.040]	0.88 [± 0.017]	0.64 [± 0.037]	0.94 [± 0.009]	0.81 [± 0.021]	0.90 [± 0.019]

Table 2. Cross-validated machine learning performance metrics of the compound models predicting 1-year overall survival.
Abbreviations: RF - random forest, XGB - extreme gradient boosting, DT - decision tree, LGR - logistic regression, kNN - k-nearest neighbour, EBM - explainable boosting machine, ACC - accuracy, SNS - sensitivity, SPC - specificity, PPV - positive predictive value, NPV - negative predictive value, BACC - balanced accuracy, AUC - area under the receiver-operator curve, CI - confidence interval

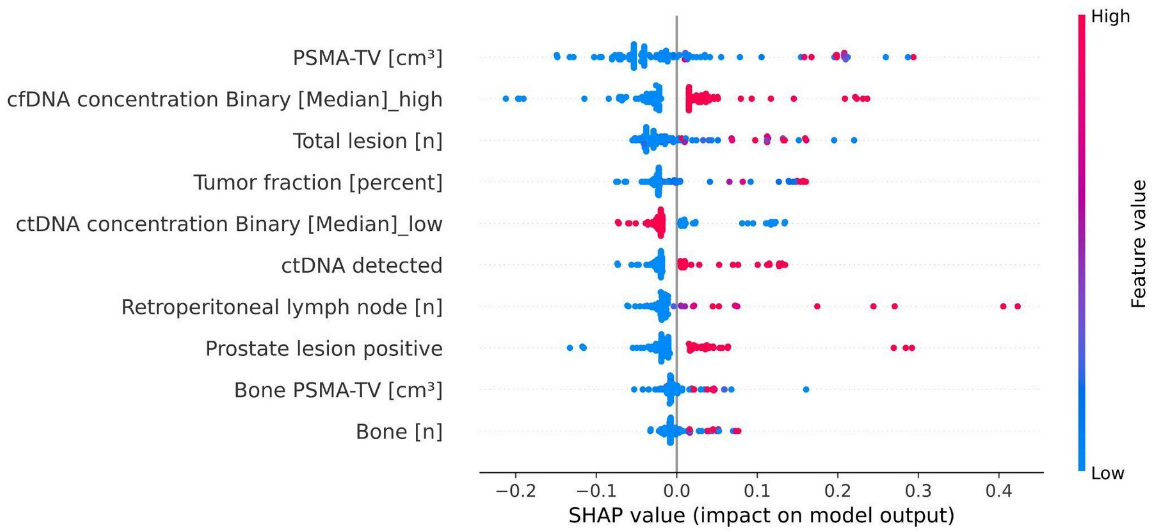

Figure 2: SHAP analysis plot illustrating the contribution and ranking of the ten most important input features to best-performing imaging model

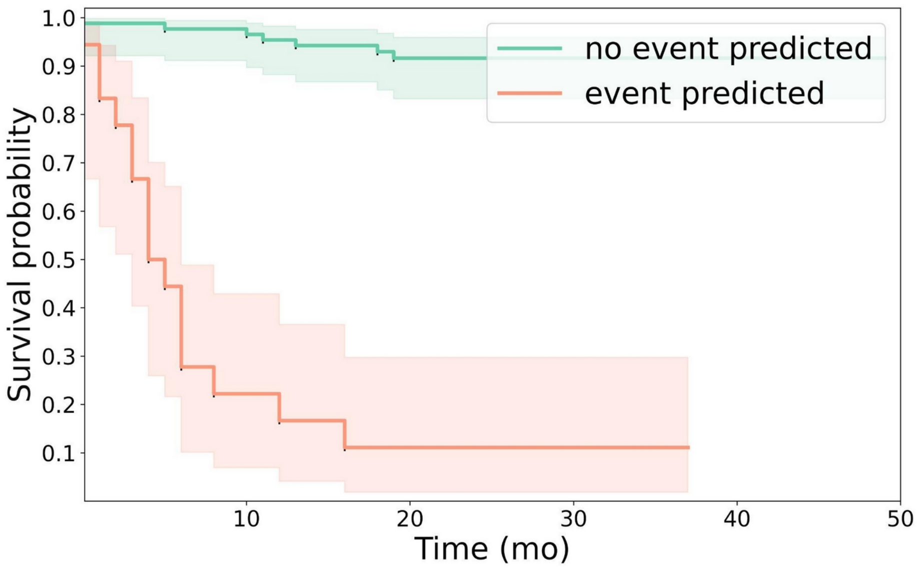

Figure 3. Kaplan-Meier curves representing the survival probabilities of groups stratified by the best-performing compound classifier

Imaging model performances and feature importances

The best-performing imaging-based classifier for the predicted endpoint 1yOS was RF. For the individual performances of all imaging-based classifiers see Table 3. For the most important features according to SHAP analysis of the best-performing model (RF) see Figure 4. For the Kaplan-Meier curves visualizing the survival probability stratification of the best-performing imaging-based classifier see Figure 5.

	ACC [± CI]	SNS [± CI]	SPC [± CI]	PPV [± CI]	NPV [± CI]	BACC [± CI]	AUC [± CI]
**RF**	0.84 [± 0.013]	0.66 [± 0.047]	0.88 [± 0.017]	0.59 [± 0.040]	0.92 [± 0.01]	0.77 [± 0.022]	0.89 [± 0.014]
**kNN**	0.81 [± 0.017]	0.65 [± 0.048]	0.84 [± 0.021]	0.53 [± 0.045]	0.91 [± 0.011]	0.74 [± 0.023]	0.82 [± 0.023]
**XGB**	0.84 [± 0.013]	0.68 [± 0.047]	0.88 [± 0.016]	0.61 [± 0.041]	0.92 [± 0.01]	0.78 [± 0.023]	0.86 [± 0.019]
**EBM**	0.83 [± 0.016]	0.73 [± 0.042]	0.85 [± 0.020]	0.58 [± 0.037]	0.93 [± 0.01]	0.79 [± 0.021]	0.88 [± 0.014]
**SVM**	0.85 [± 0.014]	0.59 [± 0.050]	0.91 [± 0.017]	0.65 [± 0.053]	0.91 [± 0.01]	0.75 [± 0.024]	0.80 [± 0.03]
**LGR**	0.84 [± 0.015]	0.69 [± 0.048]	0.88 [± 0.017]	0.60 [± 0.044]	0.93 [± 0.01]	0.78 [± 0.024]	0.84 [± 0.031]

Table 3. Cross-validated machine learning performance metrics of the imaging-based models predicting 1-year overall survival.
Abbreviations: RF - random forest, XGB - extreme gradient boosting, DT - decision tree, LGR - logistic regression, kNN - k-nearest neighbour, EBM - explainable boosting machine, ACC - accuracy, SNS - sensitivity, SPC - specificity, PPV - positive predictive value, NPV - negative predictive value, BACC - balanced accuracy, AUC - area under the receiver-operator curve, CI - confidence interval

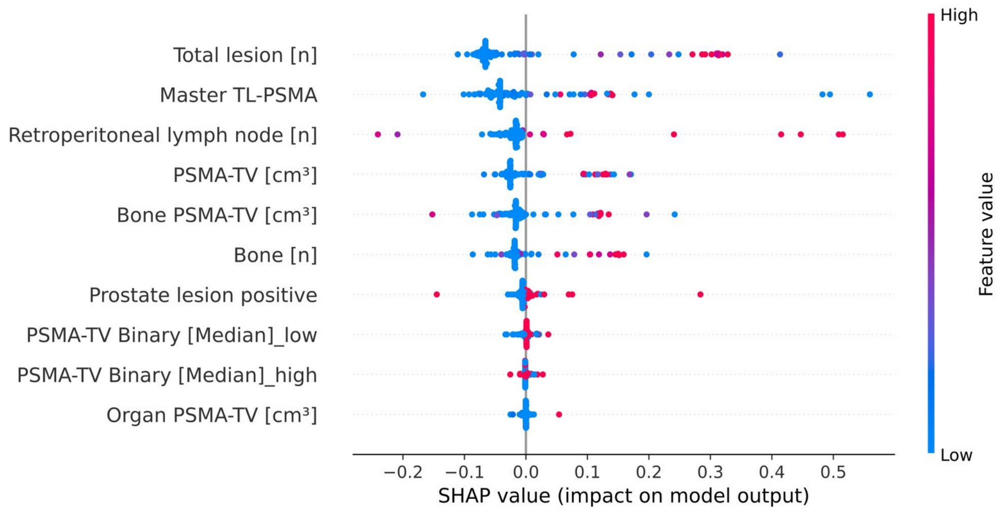

Figure 4: SHAP analysis plot illustrating the contribution and ranking of the ten most important input features to best-performing imaging model

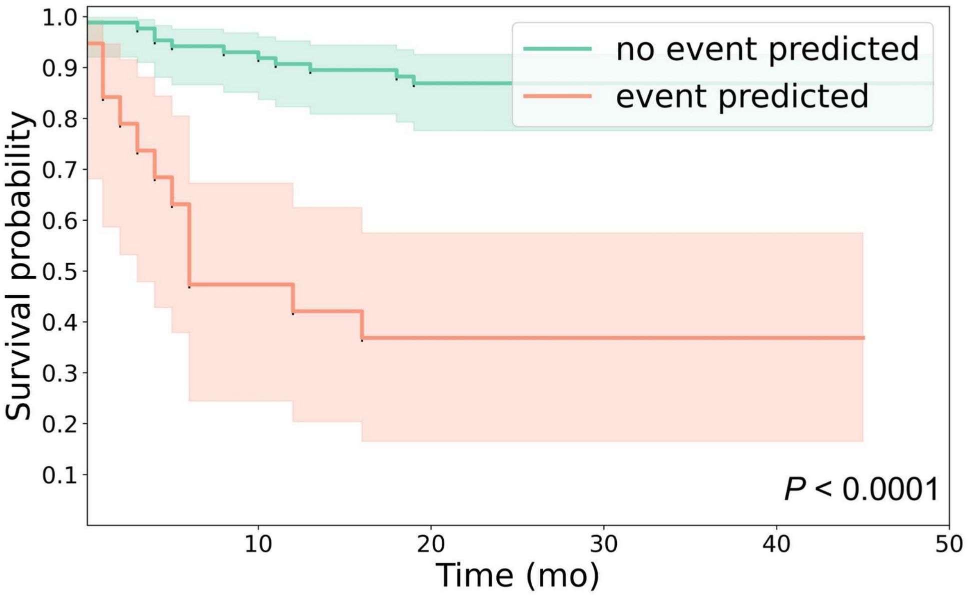

Figure 5. Kaplan-Meier curves representing the survival probabilities of groups stratified by the best-performing imaging-based classifier

Plasma model performance and feature importance

The best-performing plasma-based classifier for the predicted endpoint 1yOS was XGB. For the individual performances of all plasma-based classifiers see Table 4. For the most important features according to SHAP analysis of the best-performing model (XGB) see Figure 6. For the Kaplan-Meier curves visualizing the survival probability stratification of the best-performing plasma-based classifier see Figure 7.

	ACC [± CI]	SNS [± CI]	SPC [± CI]	PPV [± CI]	NPV [± CI]	BACC [± CI]	AUC [± CI]
**RF**	0.87 [± 0.012]	0.71 [± 0.043]	0.91 [± 0.012]	0.68 [± 0.039]	0.93 [± 0.009]	0.81 [± 0.022]	0.90 [± 0.016]
**kNN**	0.83 [± 0.015]	0.58 [± 0.048]	0.88 [± 0.016]	0.57 [± 0.049]	0.90 [± 0.01]	0.73 [± 0.025]	0.84 [± 0.022]
**XGB**	0.88 [± 0.013]	0.73 [± 0.046]	0.91 [± 0.014]	0.69 [± 0.043]	0.94 [± 0.01]	0.82 [± 0.023]	0.93 [± 0.014]
**EBM**	0.91 [± 0.01]	0.81 [± 0.035]	0.94 [± 0.01]	0.78 [± 0.032]	0.96 [± 0.01]	0.87 [± 0.018]	0.93 [± 0.012]
**SVM**	0.82 [± 0.013]	0.69 [± 0.046]	0.85 [± 0.015]	0.53 [± 0.033]	0.93 [± 0.011]	0.77 [± 0.022]	0.89 [± 0.018]
**LGR**	0.83 [± 0.014]	0.69 [± 0.047]	0.87 [± 0.016]	0.57 [± 0.037]	0.92 [± 0.011]	0.78 [± 0.023]	0.91 [± 0.015]

Table 4. Cross-validated machine learning performance metrics of the plasma-based models predicting 1-year overall survival.

Abbreviations: RF - random forest, XGB - extreme gradient boosting, DT - decision tree, LGR - logistic regression, kNN - k-nearest neighbour, EBM - explainable boosting machine, ACC - accuracy, SNS - sensitivity, SPC - specificity, PPV - positive predictive value, NPV - negative predictive value, BACC - balanced accuracy, AUC - area under the receiver-operator curve, CI - confidence interval

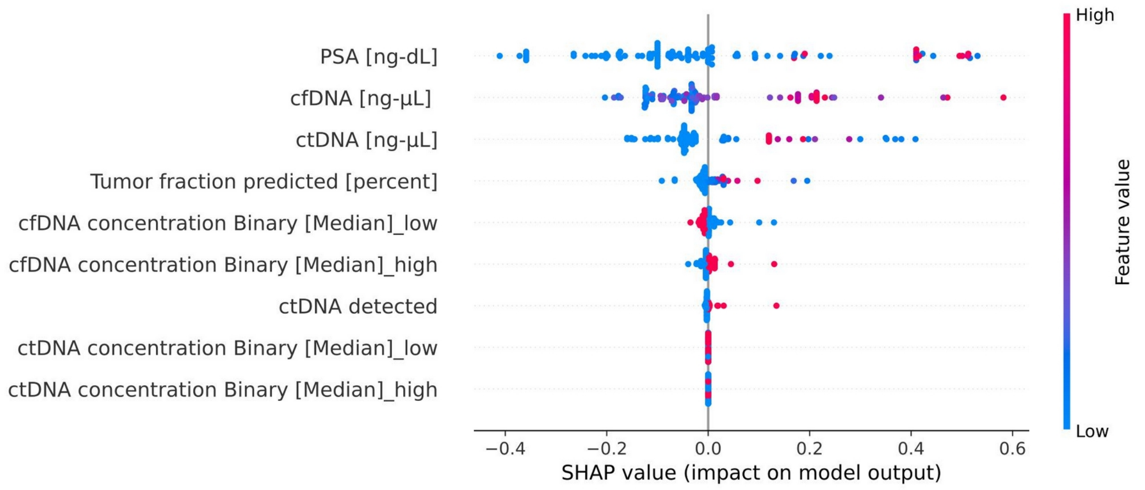

Figure 6: SHAP analysis plot illustrating the contribution and ranking of the ten most important input features to best-performing plasma model

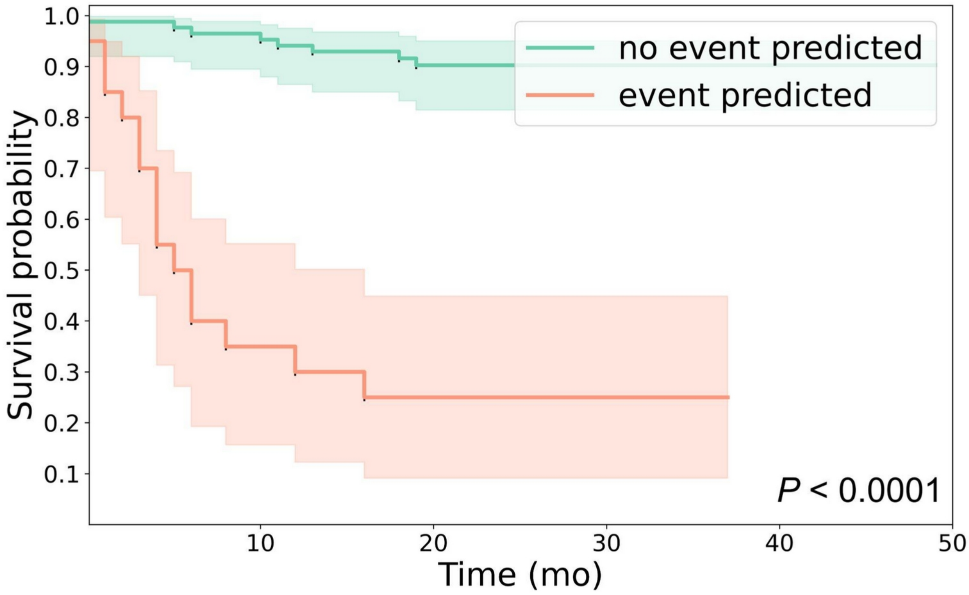

Figure 7. Kaplan-Meier curves representing the survival probabilities of groups stratified by the best-performing plasma-based classifier

Comparative overview of best-performing machine learning classifiers

	ACC [± CI]	SNS [± CI]	SPC [± CI]	PPV [± CI]	NPV [± CI]	BACC [± CI]	AUC [± CI]
$M_{compound}$	0.90 [± 0.011]	0.77 [± 0.038]	0.94 [± 0.011]	0.77 [± 0.040]	0.95 [± 0.008]	0.85 [± 0.019]	0.92 [± 0.016]
$M_{imaging}$	0.88 [± 0.012]	0.69 [± 0.044]	0.92 [± 0.013]	0.71 [± 0.042]	0.93 [± 0.009]	0.81 [± 0.022]	0.85 [± 0.023]
M_{plasma}	0.90 [± 0.013]	0.75 [± 0.043]	0.93 [± 0.011]	0.74 [± 0.040]	0.94 [± 0.009]	0.84 [± 0.022]	0.91 [± 0.020]

Table 5. Cross-validated machine learning performance metrics of the best-performing models trained on imaging- and plasma-derived (Mcompound), imaging- (Mimaging) and plasma-derived features (Mplasma) predicting 1-year overall survival
Abbreviations: ACC - accuracy, SNS - sensitivity, SPC - specificity, PPV - positive predictive value, NPV - negative predictive value, BACC - balanced accuracy, AUC - area under the receiver-operator curve, CI - confidence interval

Appendix

Feature	Definition	Compound model	Imaging model	Plasma model
cfDNA [ng/µL]	cell-free DNA concentration	x		x
cfDNA concentration Binary [Median]	median stratified binary low and high cfDNA groups	x		x
ctDNA [ng/µL]	circulating-tumor DNA concentration	x		x
ctDNA concentration Binary [Median]	median stratified binary low and high ctDNA groups	x		x
Tumor fraction predicted [%]	percentage of predicted ctDNA fraction in cfDNA	x		x
ctDNA detected	binary ctDNA detected	x		x
Dmax [mm]	maximum distance of metastasis	x	x	
Sdmax [1/m]	standardized maximum metastasis distance	x	x	
Disease extent	disease extent based on PSMA PET with the categories “no lesions”, “localized disease” comprised of prostate and seminal vesicle lesions and “metastatic”	x	x	
Prostate Lesion positive	binary any prostate lesion	x	x	
Lymphnode Lesion positive	binary any lymph node lesion	x	x	
Bone Lesion positive	binary any bone lesion	x	x	
Organ Lesion positive	binary any organ lesion	x	x	
Any Lesion positive	binary any lesion	x	x	
Prostate PSMA-TV [cm³]	PSMA-TV of prostate lesions aggregated	x	x	
Prostate SUVmin	SUVmin of prostate lesions aggregated	x	x	
Prostate SUVmax	SUVmax of prostate lesions aggregated	x	x	
Prostate SUVmean	SUVmean of prostate lesions aggregated	x	x	
Prostate SUVpeak	SUVpeak of prostate lesions aggregated	x	x	
Prostate TL-PSMA	Total lesion PSMA-TV of prostate lesions aggregated	x	x	
Lymph node PSMA-TV [cm³]	PSMA-TV of lymph node lesions aggregated	x	x	
Lymph node SUVmin	SUVmin of lymph node lesions aggregated	x	x	

Lymph node SUVmax	SUVmax of lymph node lesions aggregated	x	x
Lymph node SUVmean	SUVmean of lymph node lesions aggregated	x	x
Lymph node SUVpeak	SUVpeak of lymph node lesions aggregated	x	x
Lymph node TL-PSMA	Total lesion PSMA-TV of lymph node lesions aggregated	x	x
Bone PSMA-TV [cm³]	PSMA-TV of bone lesions aggregated	x	x
Bone SUVmin	SUVmin of bone lesions aggregated	x	x
Bone SUVmax	SUVmax of bone lesions aggregated	x	x
Bone SUVmean	SUVmean of bone lesions aggregated	x	x
Bone SUVpeak	SUVpeak of bone lesions aggregated	x	x
Bone TL-PSMA	Total lesion PSMA-TV of bone lesions aggregated	x	x
Organ PSMA-TV [cm³]	PSMA-TV of organ lesions aggregated	x	x
Organ SUVmin	SUVmin of organ lesions aggregated	x	x
Organ SUVmax	SUVmax of organ lesions aggregated	x	x
Organ SUVmean	SUVmean of organ lesions aggregated	x	x
Organ SUVpeak	SUVpeak of organ lesions aggregated	x	x
Organ TL-PSMA	Total lesion PSMA-TV of organ lesions aggregated	x	x
PSMA-TV [cm³]	PSMA-TV of all lesions aggregated	x	x
PSMA-TV Binary [Median]	median stratified binary PSMA-TV of all lesions	x	x
Master SUVmin	SUVmin of all lesions aggregated	x	x
Master SUVmax	SUVmax of all lesions aggregated	x	x
Master SUVmean	SUVmean of all lesions aggregated	x	x
Master SUVpeak	SUVpeak of all lesions aggregated	x	x
Master TL-PSMA	Total lesion PSMA-TV of all lesions aggregated	x	x
PSMA-TV local fraction [%]	percentage of total PSMA-TV contributed by prostate lesions	x	x
PSMA-TV lymph node fraction [%]	percentage of total PSMA-TV contributed by lymph node lesions	x	x
PSMA-TV bone fraction [%]	percentage of total PSMA-TV contributed by bone lesions	x	x
PSMA-TV organ fraction [%]	percentage of total PSMA-TV contributed by organ lesions	x	x
Dominant fraction	lesion fraction contributing most to total PSMA-TV	x	x
Total lesion [n]	total number of lesions	x	x
Total prostate or seminal vesicle lesion [n]	total number of lesions in prostate or seminal vesicles	x	x

Prostate [n]	total number of prostate lesions	x	x	
Pelvic lymph nodes [n]	total number of pelvic lymph node lesions	x	x	
Retroperitoneal lymph node [n]	total number of retroperitoneal lymph node lesions	x	x	
Distant lymph node [n]	total number of distant lymph node lesions	x	x	
Bone [n]	total number of bone lesions	x	x	
Total organ [n]	total number of organ lesions	x	x	
PSA [ng/mL]	plasma prostate-specific antigen level	x		x

Table 6. Input features used in the compound, imaging and plasma-based models

References

1. Scher HI, Halabi S, Tannock I, Morris M, Sternberg CN, Carducci MA, et al. Design and end points of clinical trials for patients with progressive prostate cancer and castrate levels of testosterone: recommendations of the Prostate Cancer Clinical Trials Working Group. J Clin Oncol. 2008;26:1148–59.

2. Li H, Durbin R. Fast and accurate short read alignment with Burrows-Wheeler transform. Bioinformatics. 2009;25:1754–60.

3. Adalsteinsson VA, Ha G, Freeman SS, Choudhury AD, Stover DG, Parsons HA, et al. Scalable whole-exome sequencing of cell-free DNA reveals high concordance with metastatic tumors. Nat Commun. 2017;8:1324.

4. Peng H, Long F, Ding C. Feature selection based on mutual information: criteria of max-dependency, max-relevance, and min-redundancy. IEEE Trans Pattern Anal Mach Intell. 2005;27:1226–38.

5. Chawla NV, Bowyer KW, Hall LO, Kegelmeyer WP. SMOTE: Synthetic Minority Over-sampling Technique. jair. 2002;16:321–57.

6. Nori H, Jenkins S, Koch P, Caruana R. InterpretML: A Unified Framework for Machine Learning Interpretability [Internet]. arXiv [cs.LG]. 2019. Available from: http://arxiv.org/abs/1909.09223

7. Lundberg SM, Erion GG, Lee S-I. Consistent Individualized Feature Attribution for Tree Ensembles [Internet]. arXiv [cs.LG]. 2018. Available from: http://arxiv.org/abs/1802.03888

8. Harris CR, Millman KJ, van der Walt SJ, Gommers R, Virtanen P, Cournapeau D, et al. Array programming with NumPy. Nature. 2020;585:357–62.

9. Mc Kinney W. Pandas: A foundational python library for data analysis and statistics [Internet]. [cited 2023 Sep 4]. Available from: https://www.dlr.de/sc/portaldata/15/resources/dokumente/pyhpc2011/submissions/pyhpc2011_submission_9.pdf

10. Waskom M. seaborn: statistical data visualization. J Open Source Softw. 2021;6:3021.

11. Hunter. Matplotlib: A 2D Graphics Environment. 2007;9:90–5.

12. Pedregosa F, Varoquaux G, Gramfort A, Michel V. Scikit-learn: Machine Learning in Python. arXiv [cs. LG]. 2012. is no corresponding record for this ….

13. Lemaitre G, Nogueira F, Aridas CK. Imbalanced-learn: A python toolbox to tackle the curse of imbalanced datasets in machine learning [Internet]. arXiv [cs.LG]. 2016 [cited 2023 Sep 4]. Available from: https://www.jmlr.org/papers/volume18/16-365/16-365.pdf

14. Lundberg SM, Erion G, Chen H, DeGrave A, Prutkin JM, Nair B, et al. From Local Explanations to Global Understanding with Explainable AI for Trees. Nat Mach Intell. 2020;2:56–67.

15. Chen T, Guestrin C. XGBoost: A Scalable Tree Boosting System. Proceedings of the 22nd ACM SIGKDD International Conference on Knowledge Discovery and Data Mining.

New York, NY, USA: Association for Computing Machinery; 2016. p. 785–94.

Example sequencing reports and statistics

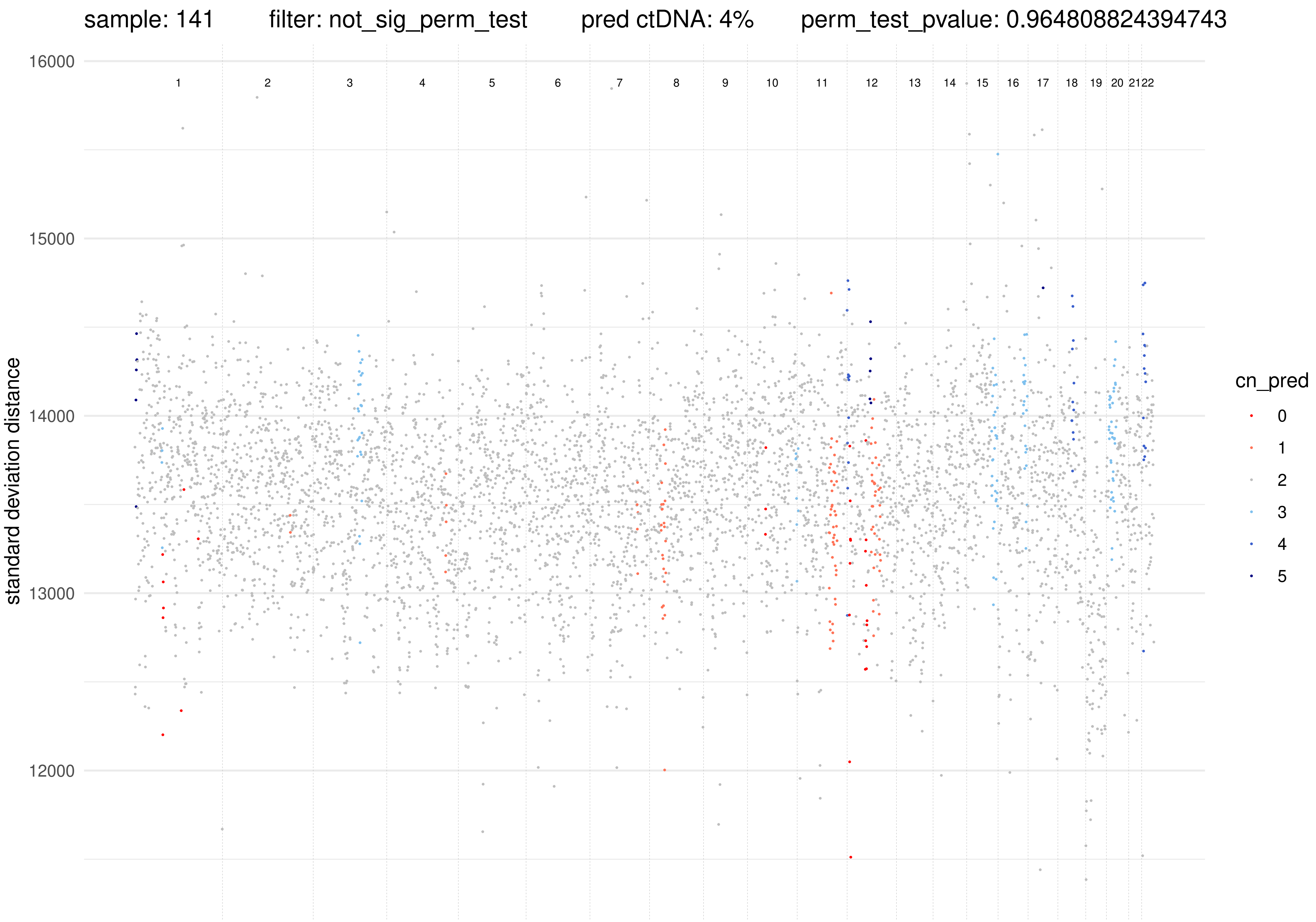

sample: 141
filter: not_sig_perm_test
pred ctDNA: 4%
perm_test_pvalue: 0.964808824394743
standard deviation distance
16000
15000
14000
13000
12000
1
2
3
4
5
6
7
8
9
10
11
12
13
14
15
16
17
18
19
20
21
22
cn_pred
0
1
2
3
4
5

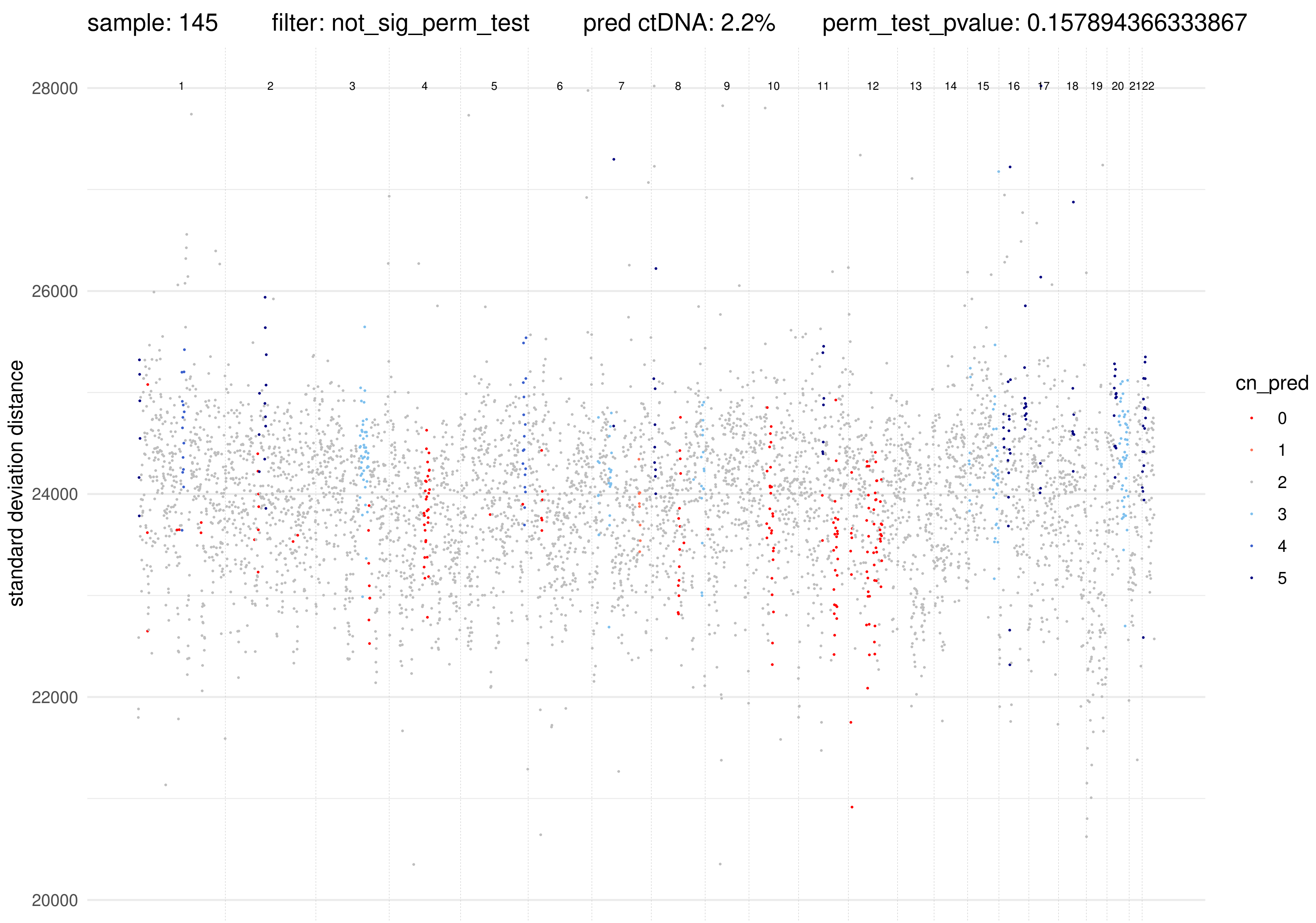

sample: 145
filter: not_sig_perm_test
pred ctDNA: 2.2%
perm_test_pvalue: 0.157894366333867
standard deviation distance
28000
26000
24000
22000
20000
1
2
3
4
5
6
7
8
9
10
11
12
13
14
15
16
17
18
19
20
21
22
cn_pred
0
1
2
3
4
5

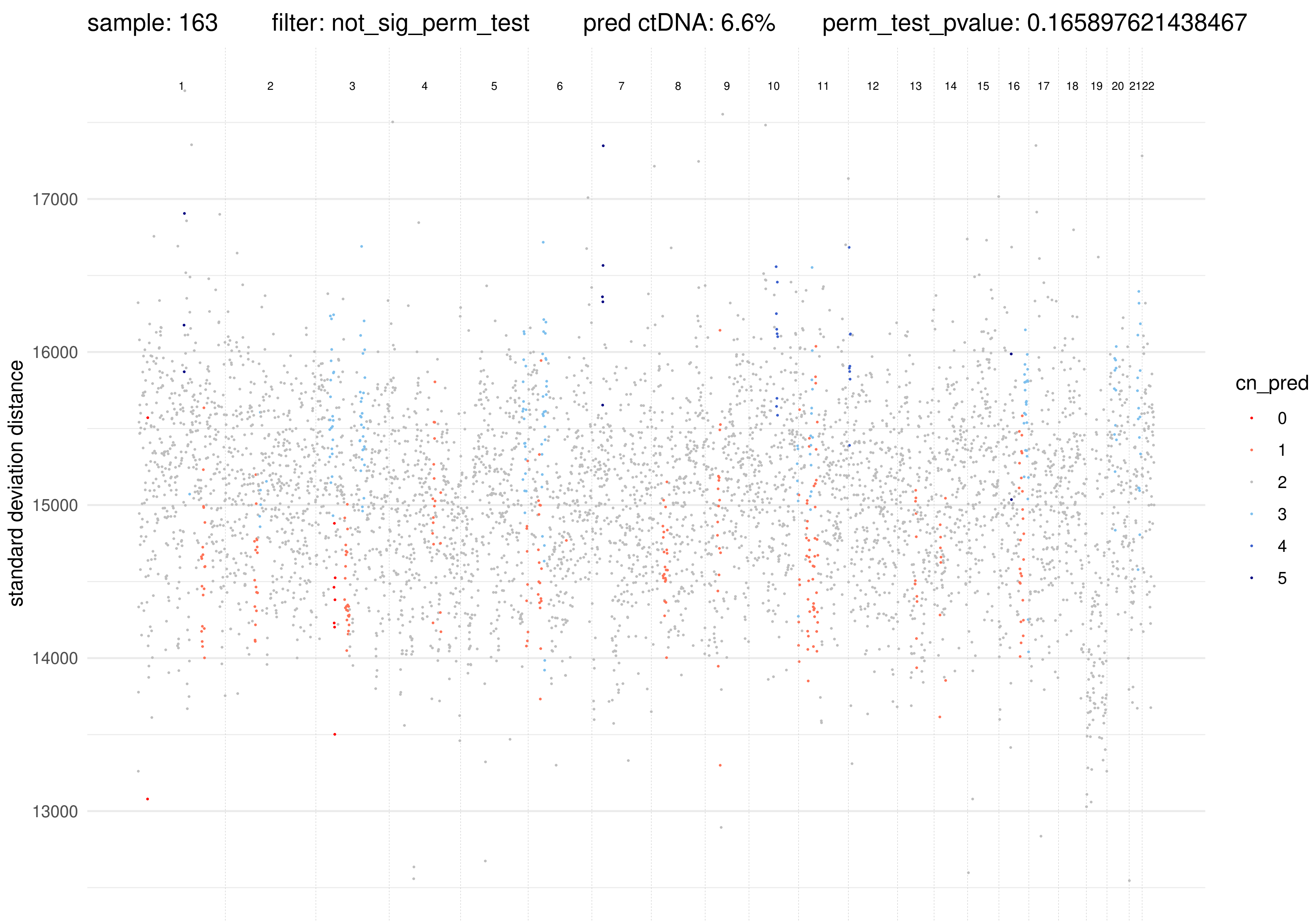
sample: 163 filter: not_sig_perm_test pred ctDNA: 6.6% perm_test_pvalue: 0.165897621438467
1
2
3
4
5
6
7
8
9
10
11
12
13
14
15
16
17
18
19
20
21
22
standard deviation distance
17000
16000
15000
14000
13000
cn_pred
0
1
2
3
4
5

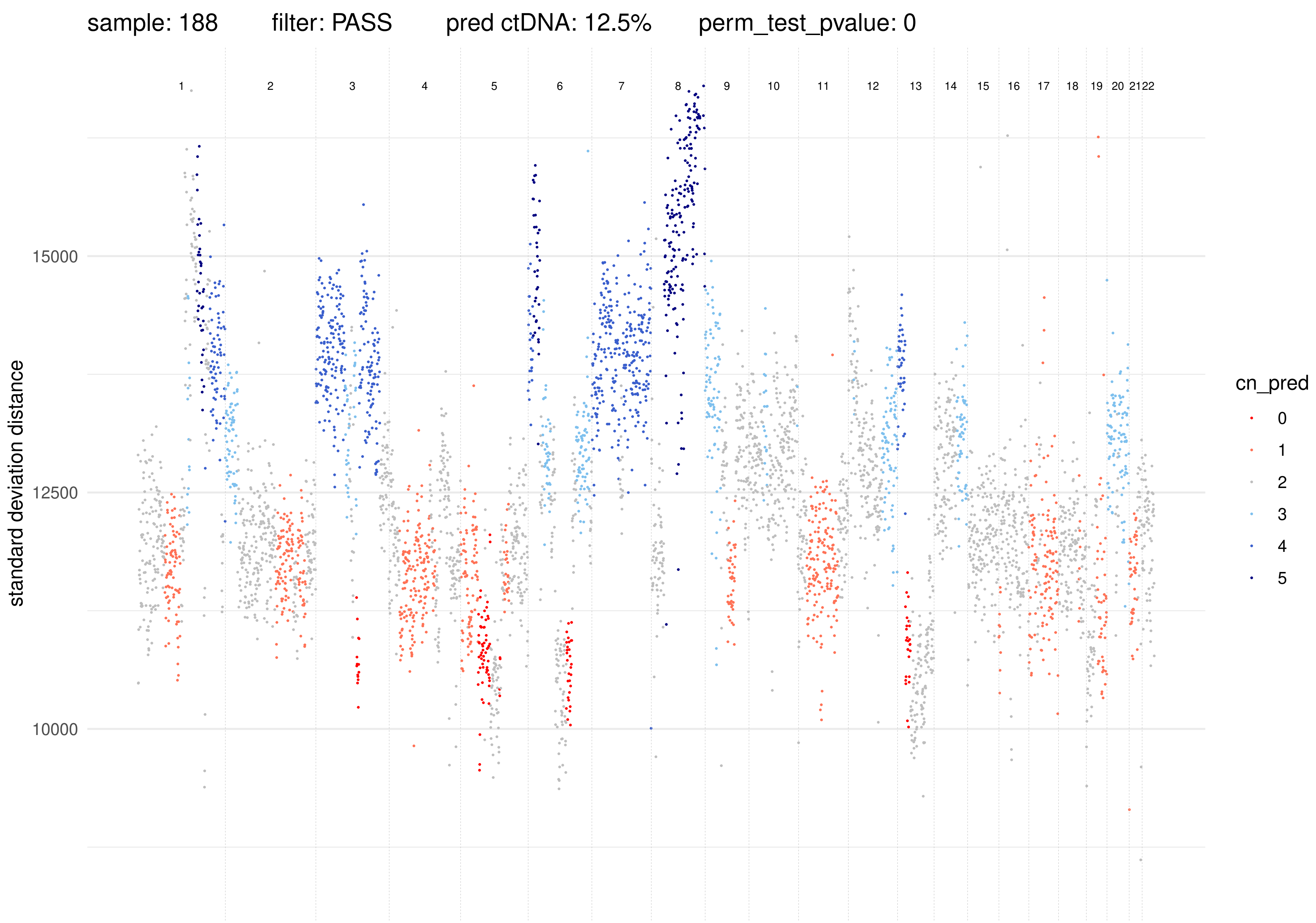

sample: 188
filter: PASS
pred ctDNA: 12.5%
perm_test_pvalue: 0
1
2
3
4
5
6
7
8
9
10
11
12
13
14
15
16
17
18
19
20
21
22
15000
12500
10000
standard deviation distance
cn_pred
0
1
2
3
4
5

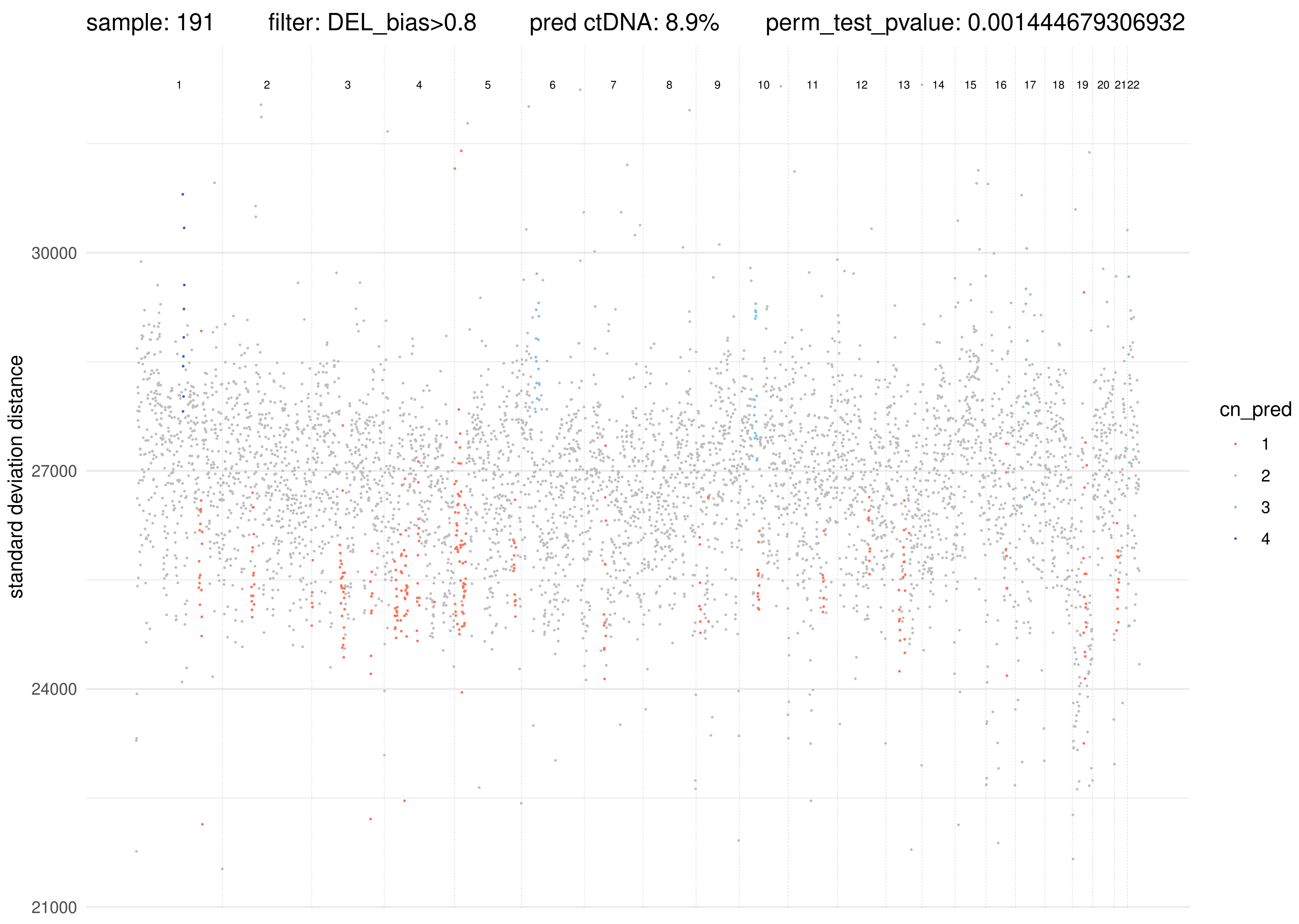
sample: 191
filter: DEL_bias>0.8
pred ctDNA: 8.9%
perm_test_pvalue: 0.001444679306932
1
2
3
4
5
6
7
8
9
10
11
12
13
14
15
16
17
18
19
20
21
22
standard deviation distance
30000
27000
24000
21000
cn_pred
1
2
3
4

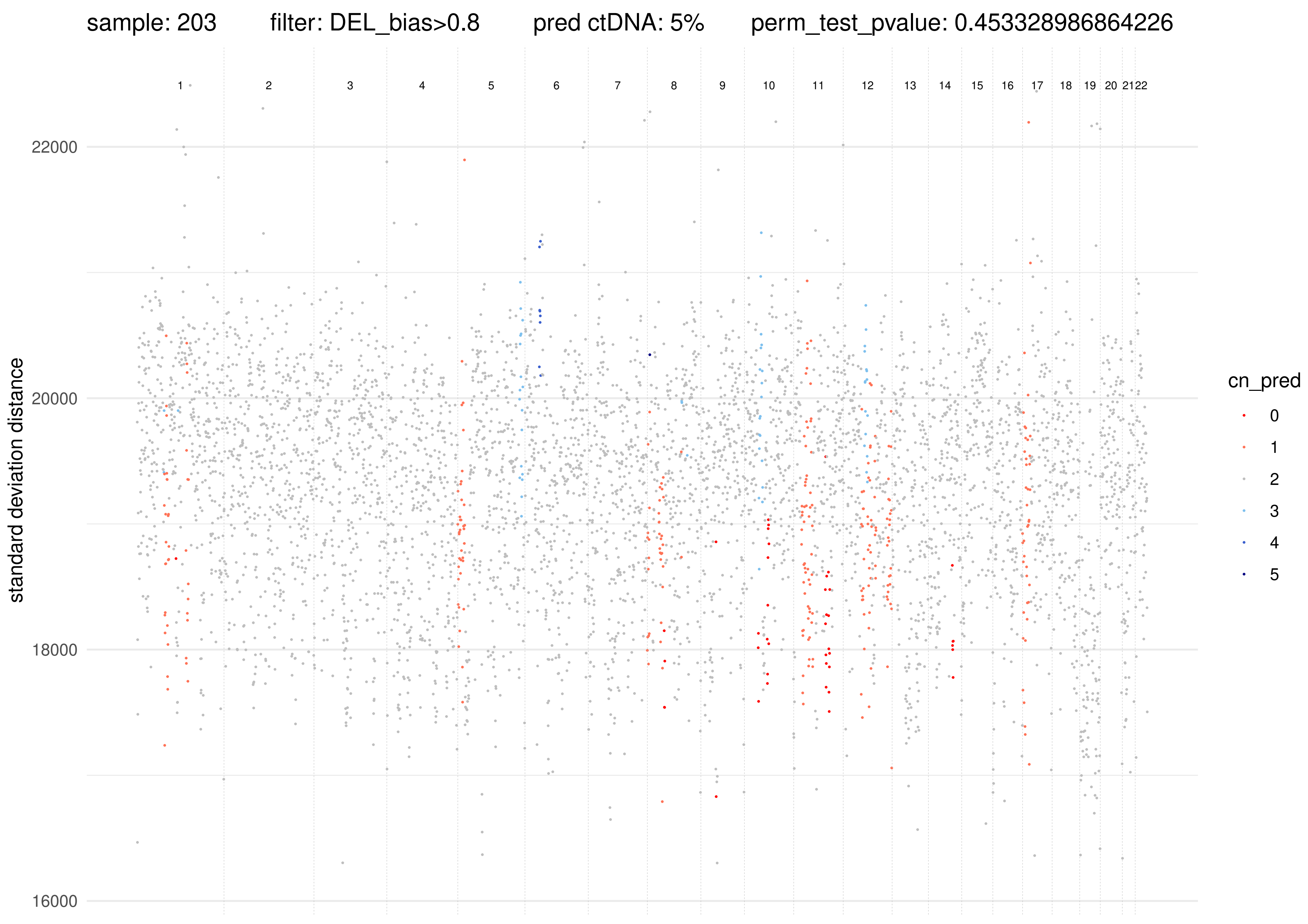
sample: 203
filter: DEL_bias>0.8
pred ctDNA: 5%
perm_test_pvalue: 0.453328986864226
1
2
3
4
5
6
7
8
9
10
11
12
13
14
15
16
17
18
19
20
21
22
standard deviation distance
22000
20000
18000
16000
cn_pred
0
1
2
3
4
5

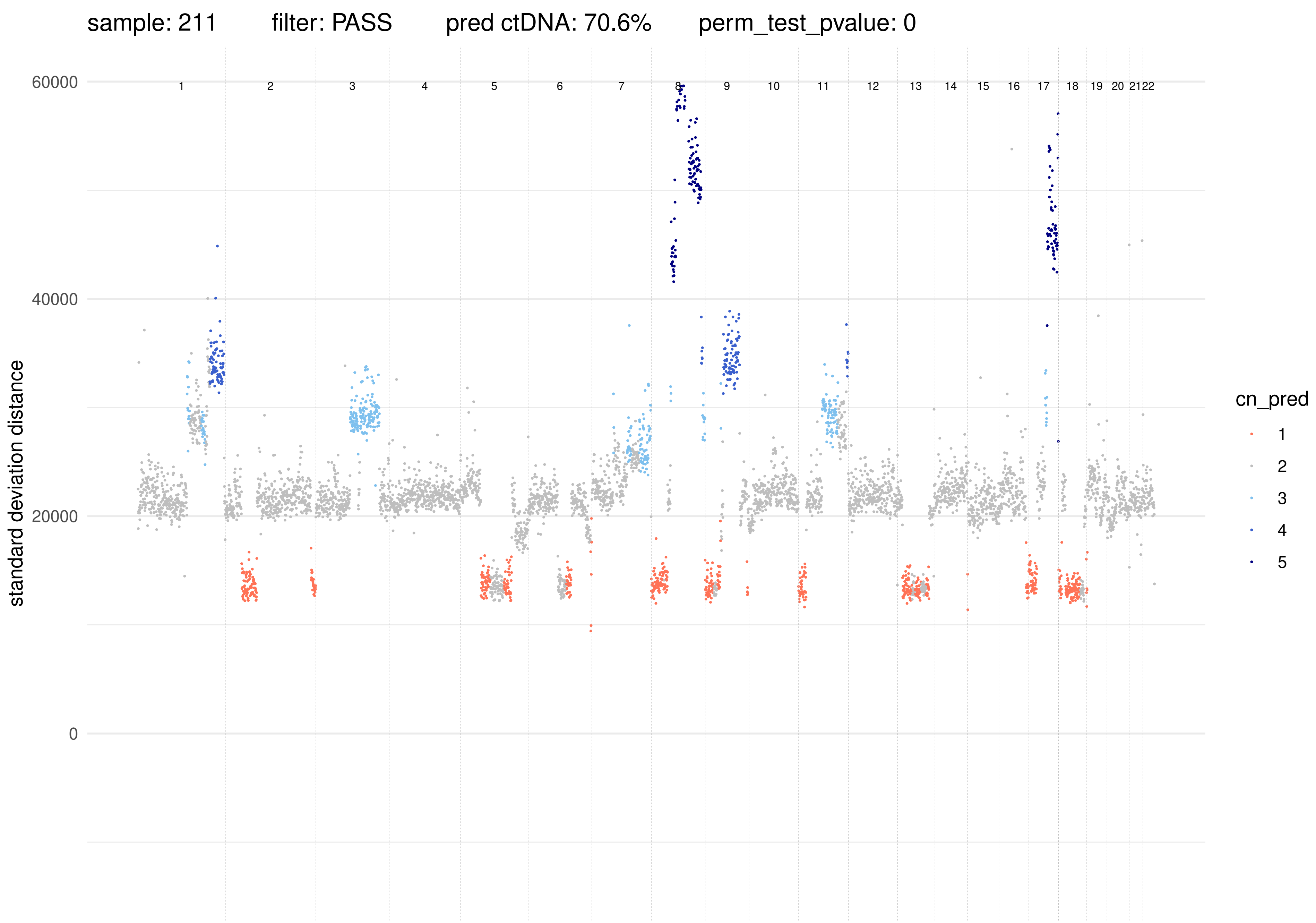
sample: 211
filter: PASS
pred ctDNA: 70.6%
perm_test_pvalue: 0
standard deviation distance
60000
40000
20000
0
1
2
3
4
5
6
7
8
9
10
11
12
13
14
15
16
17
18
19
20
21
22
cn_pred
1
2
3
4
5

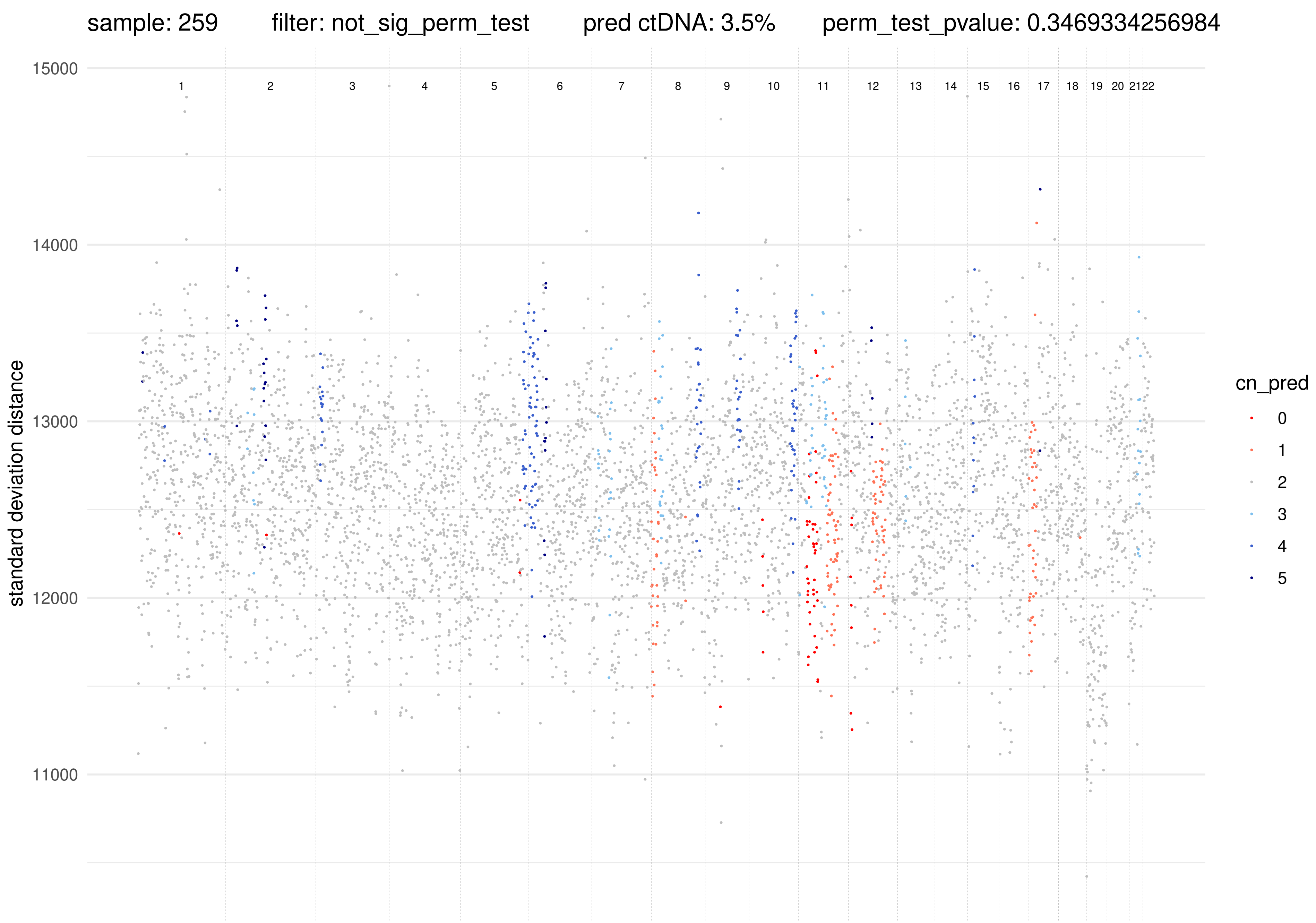

sample: 259
filter: not_sig_perm_test
pred ctDNA: 3.5%
perm_test_pvalue: 0.3469334256984
standard deviation distance
15000
14000
13000
12000
11000
1
2
3
4
5
6
7
8
9
10
11
12
13
14
15
16
17
18
19
20
21
22
cn_pred
0
1
2
3
4
5

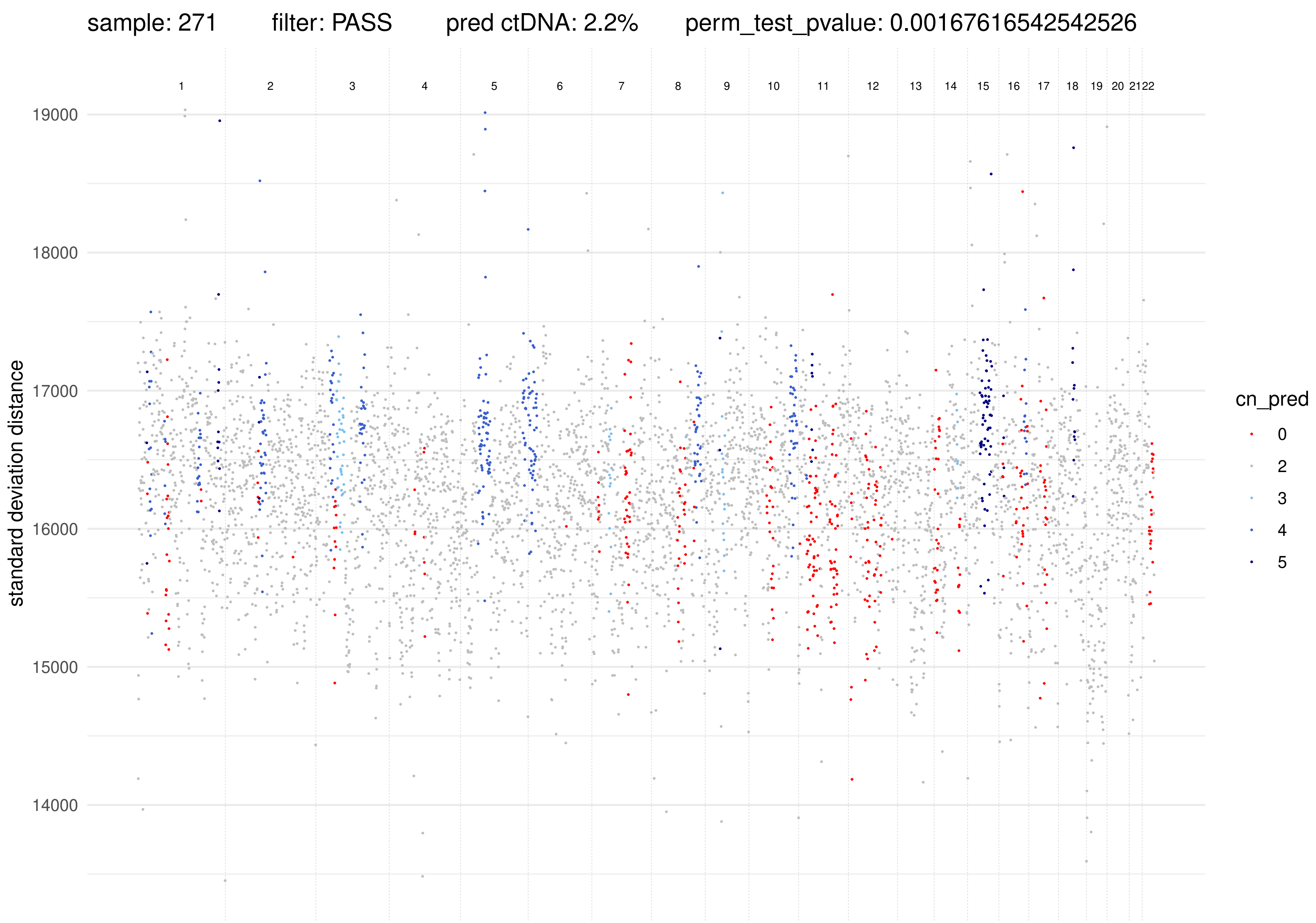
sample: 271
filter: PASS
pred ctDNA: 2.2%
perm_test_pvalue: 0.00167616542542526
1
2
3
4
5
6
7
8
9
10
11
12
13
14
15
16
17
18
19
20
21
22
19000
18000
17000
16000
15000
14000
standard deviation distance
cn_pred
0
2
3
4
5

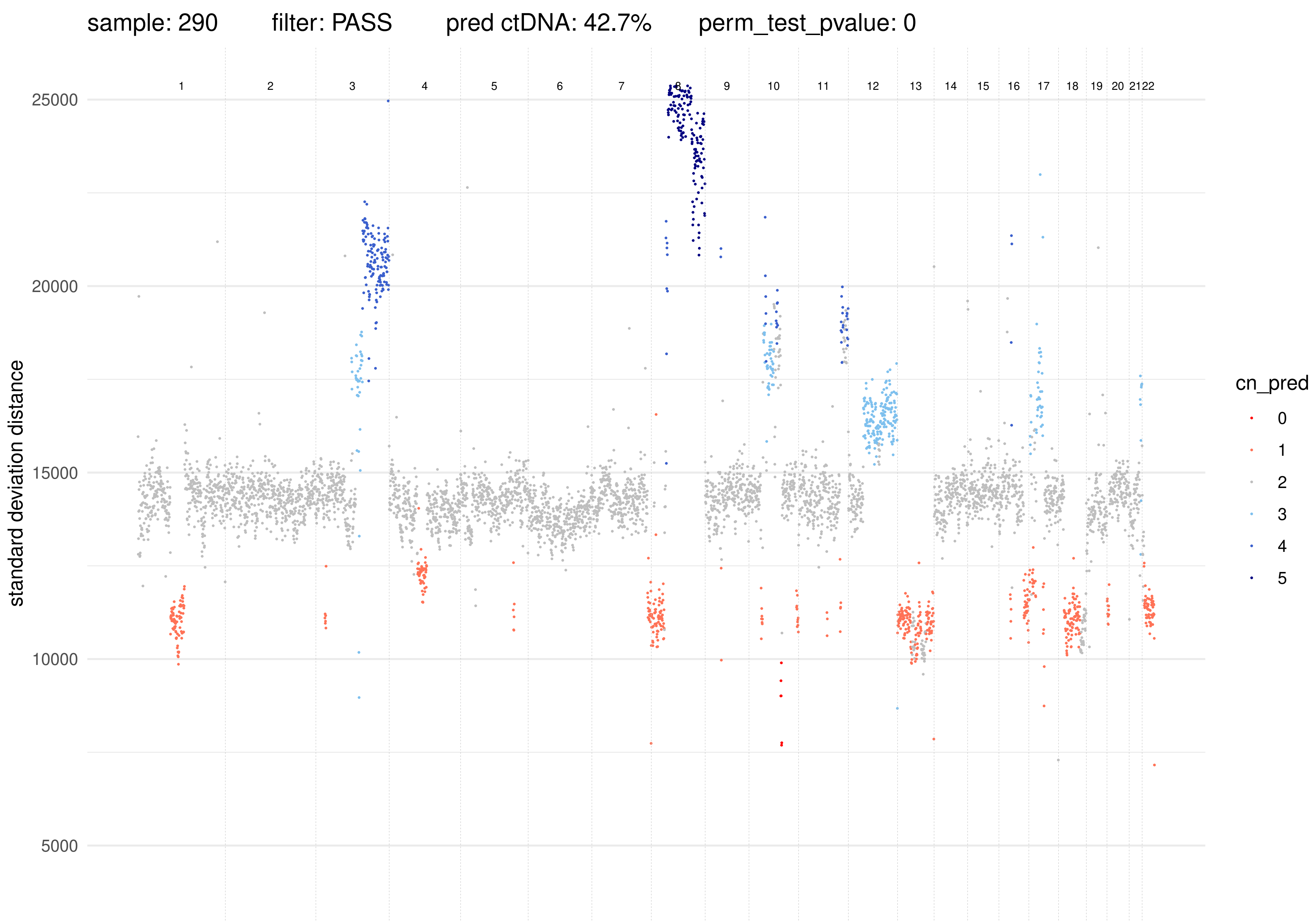
sample: 290
filter: PASS
pred ctDNA: 42.7%
perm_test_pvalue: 0
1
2
3
4
5
6
7
8
9
10
11
12
13
14
15
16
17
18
19
20
21
22
25000
20000
15000
10000
5000
standard deviation distance
cn_pred
0
1
2
3
4
5

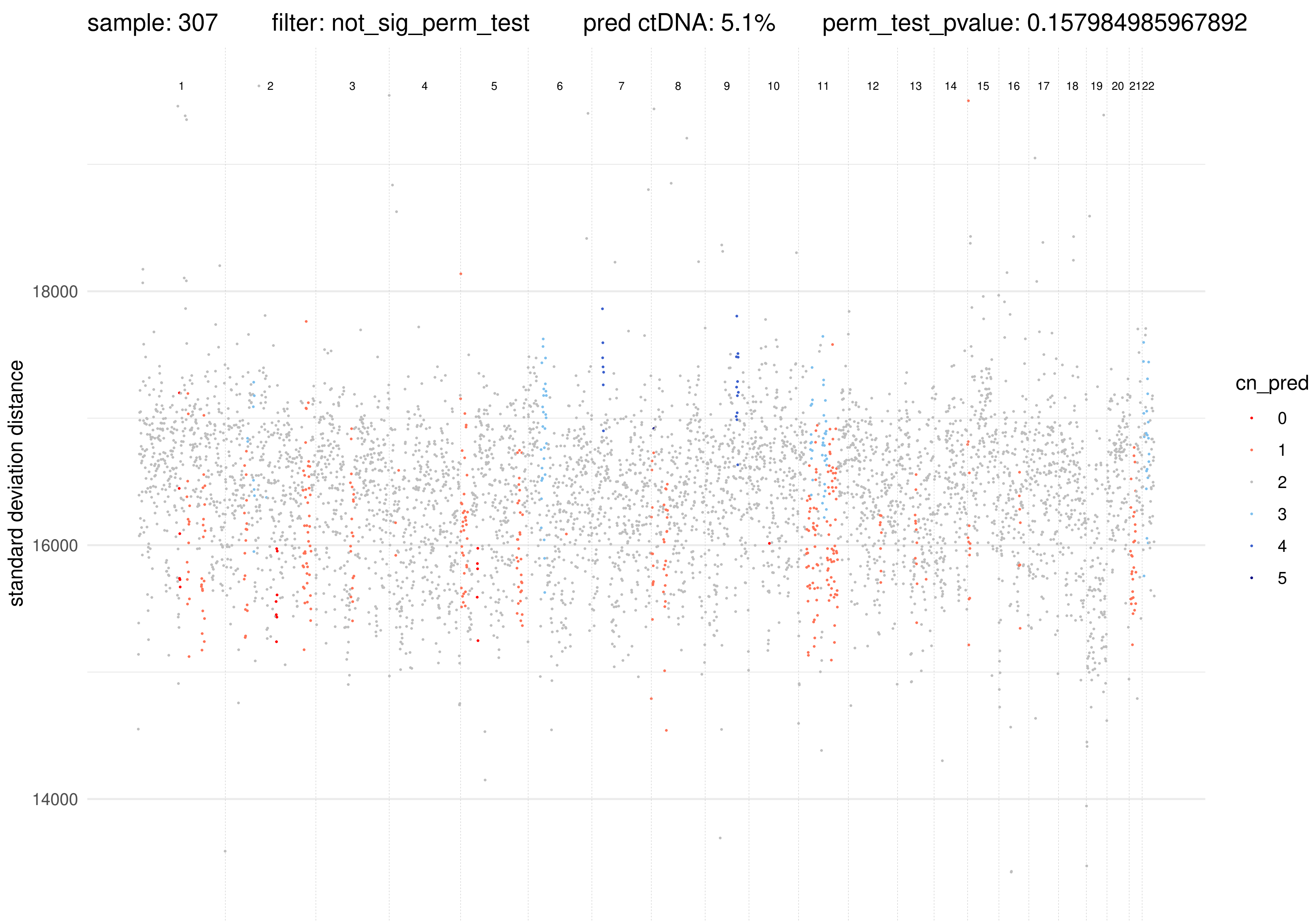
sample: 307 filter: not_sig_perm_test pred ctDNA: 5.1% perm_test_pvalue: 0.157984985967892
1
2
3
4
5
6
7
8
9
10
11
12
13
14
15
16
17
18
19
20
21
22
standard deviation distance
18000
16000
14000
cn_pred
0
1
2
3
4
5

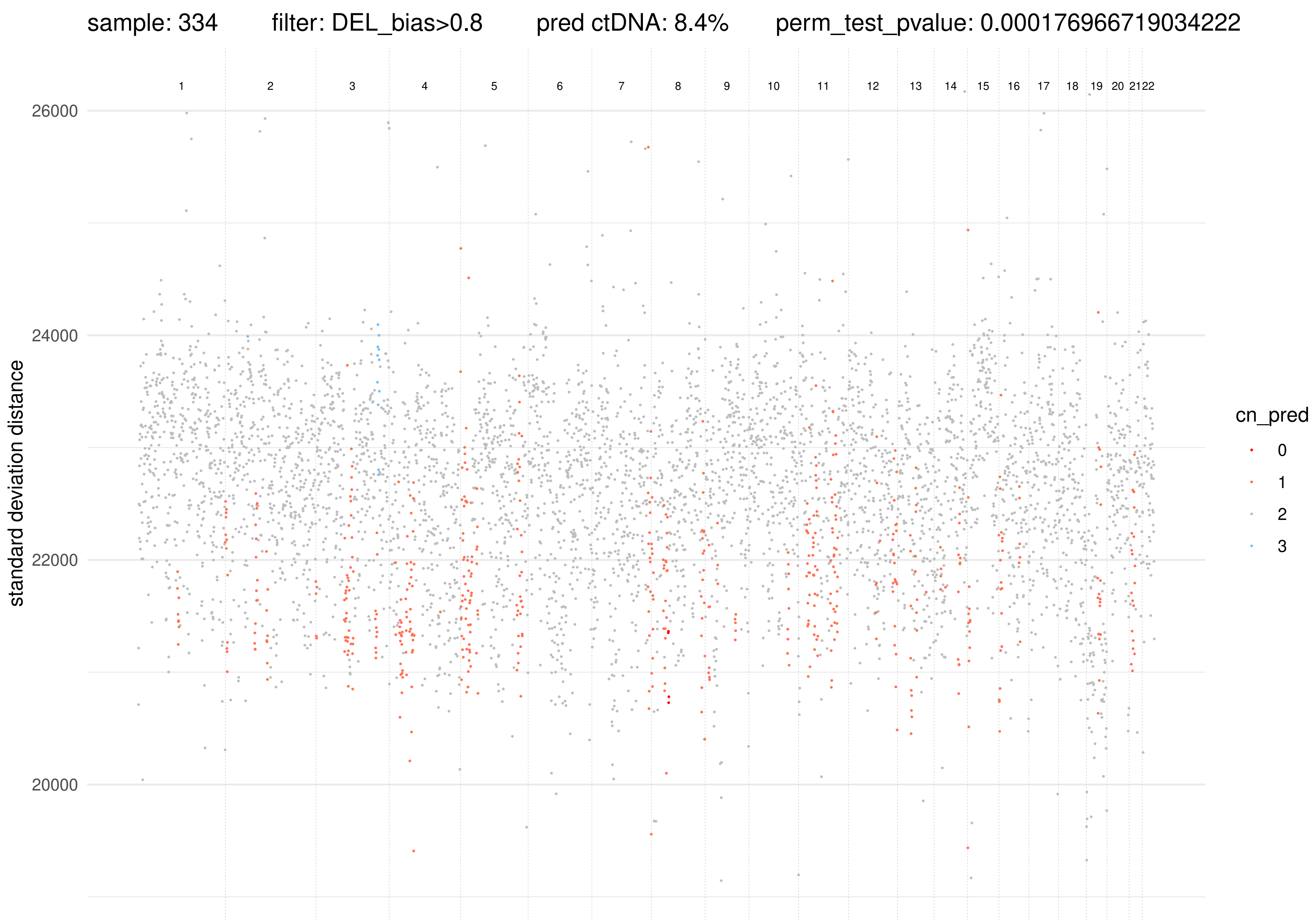

sample: 334
filter: DEL_bias>0.8
pred ctDNA: 8.4%
perm_test_pvalue: 0.000176966719034222
1
2
3
4
5
6
7
8
9
10
11
12
13
14
15
16
17
18
19
20
21
22
26000
24000
22000
20000
standard deviation distance
cn_pred
0
1
2
3

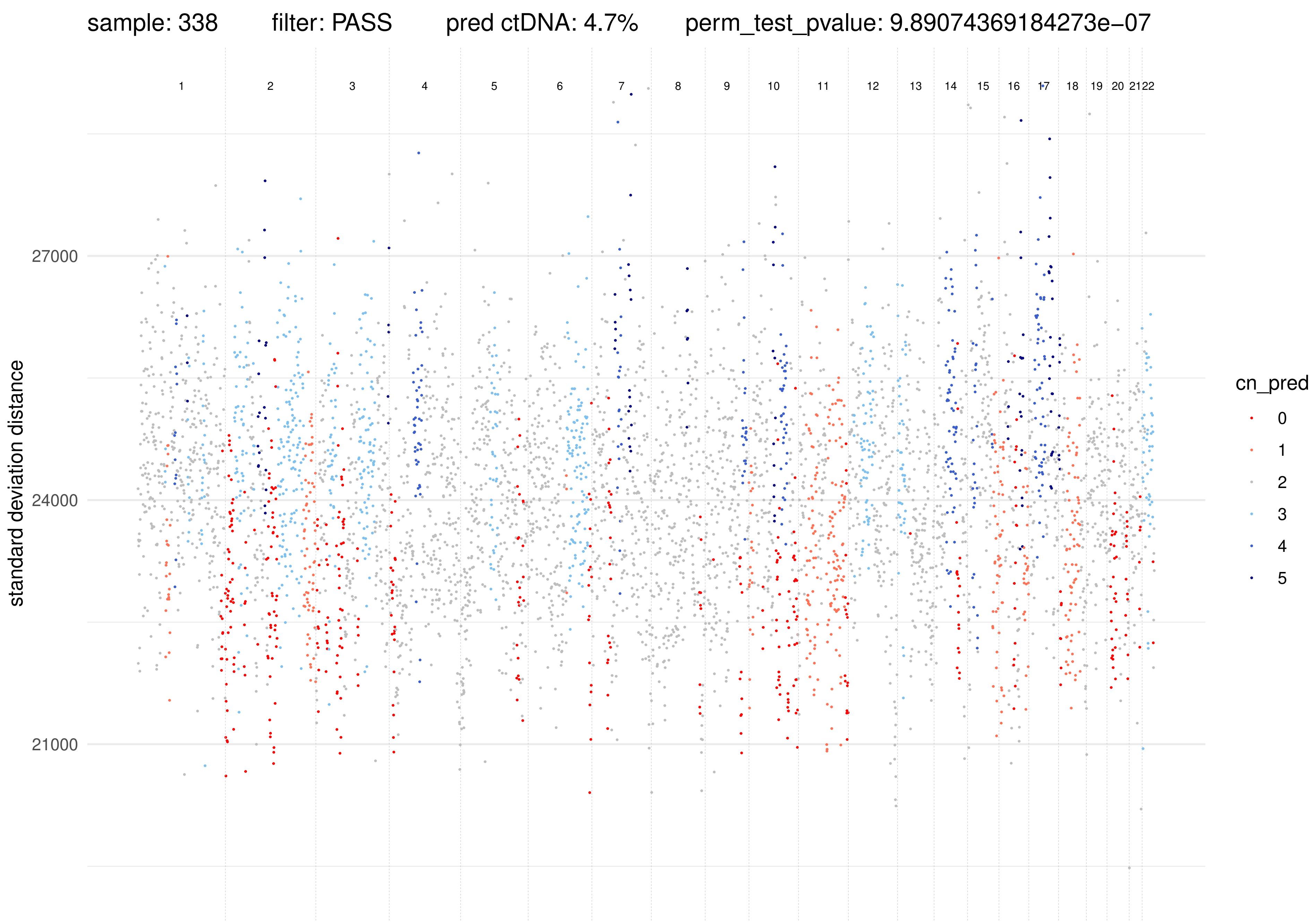

sample: 338
filter: PASS
pred ctDNA: 4.7%
perm_test_pvalue: 9.89074369184273e−07
1
2
3
4
5
6
7
8
9
10
11
12
13
14
15
16
17
18
19
20
21
22
standard deviation distance
27000
24000
21000
cn_pred
0
1
2
3
4
5

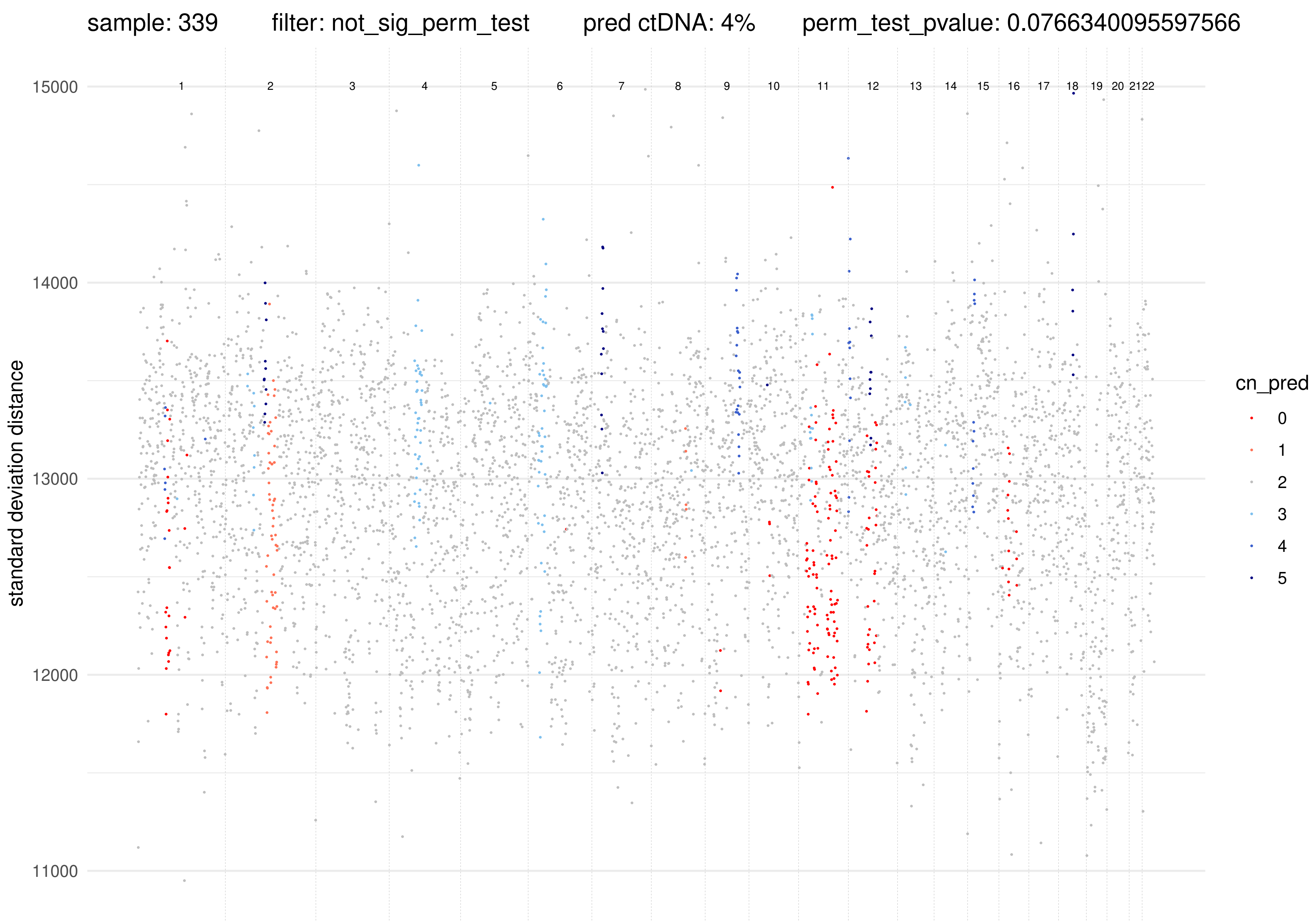
sample: 339
filter: not_sig_perm_test
pred ctDNA: 4%
perm_test_pvalue: 0.0766340095597566
standard deviation distance
15000
14000
13000
12000
11000
1
2
3
4
5
6
7
8
9
10
11
12
13
14
15
16
17
18
19
20
21
22
cn_pred
0
1
2
3
4
5

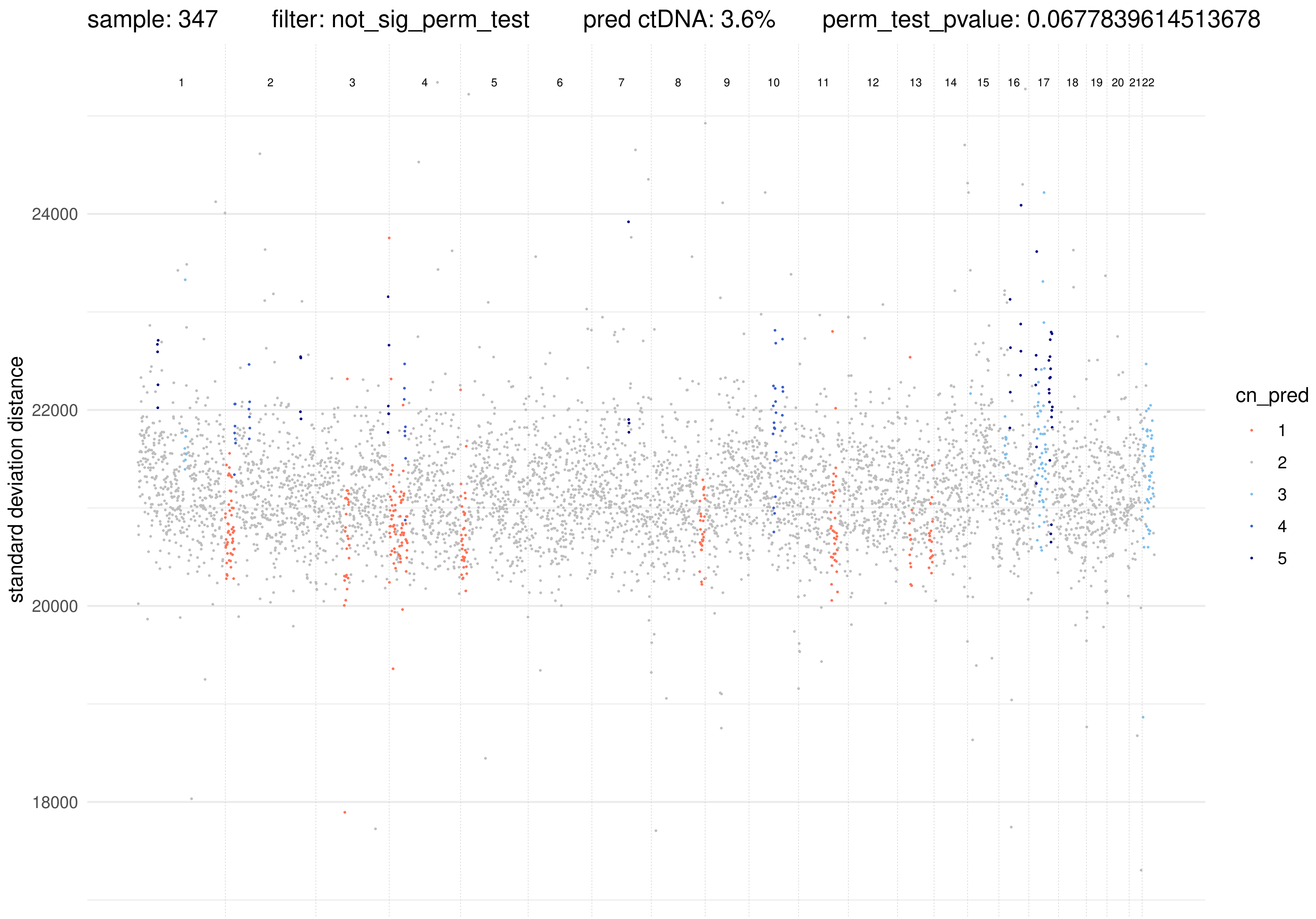
sample: 347
filter: not_sig_perm_test
pred ctDNA: 3.6%
perm_test_pvalue: 0.0677839614513678
1
2
3
4
5
6
7
8
9
10
11
12
13
14
15
16
17
18
19
20
21
22
standard deviation distance
24000
22000
20000
18000
cn_pred
1
2
3
4
5

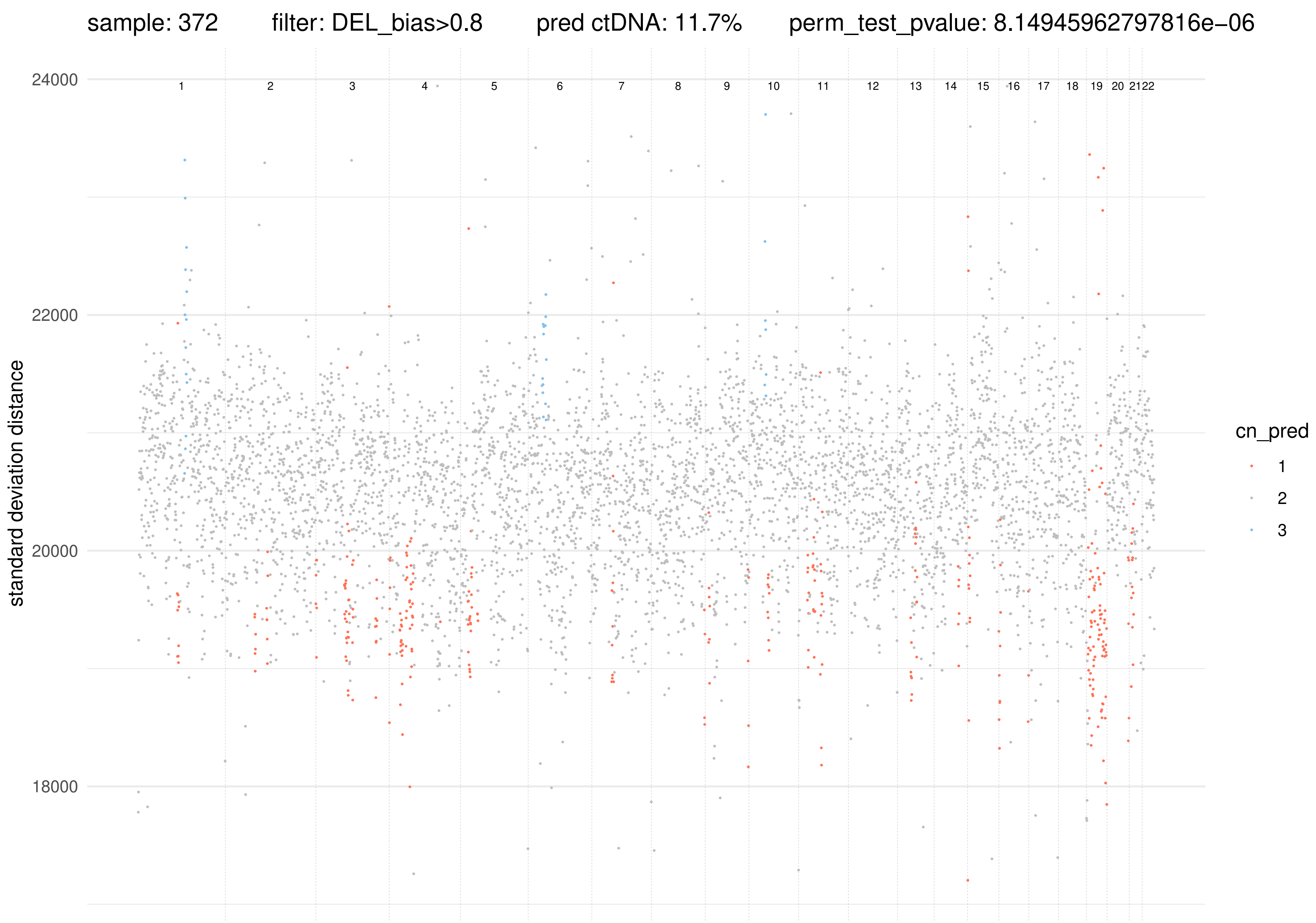

sample: 372
filter: DEL_bias>0.8
pred ctDNA: 11.7%
perm_test_pvalue: 8.14945962797816e−06
standard deviation distance
24000
22000
20000
18000
1
2
3
4
5
6
7
8
9
10
11
12
13
14
15
16
17
18
19
20
21
22
cn_pred
1
2
3

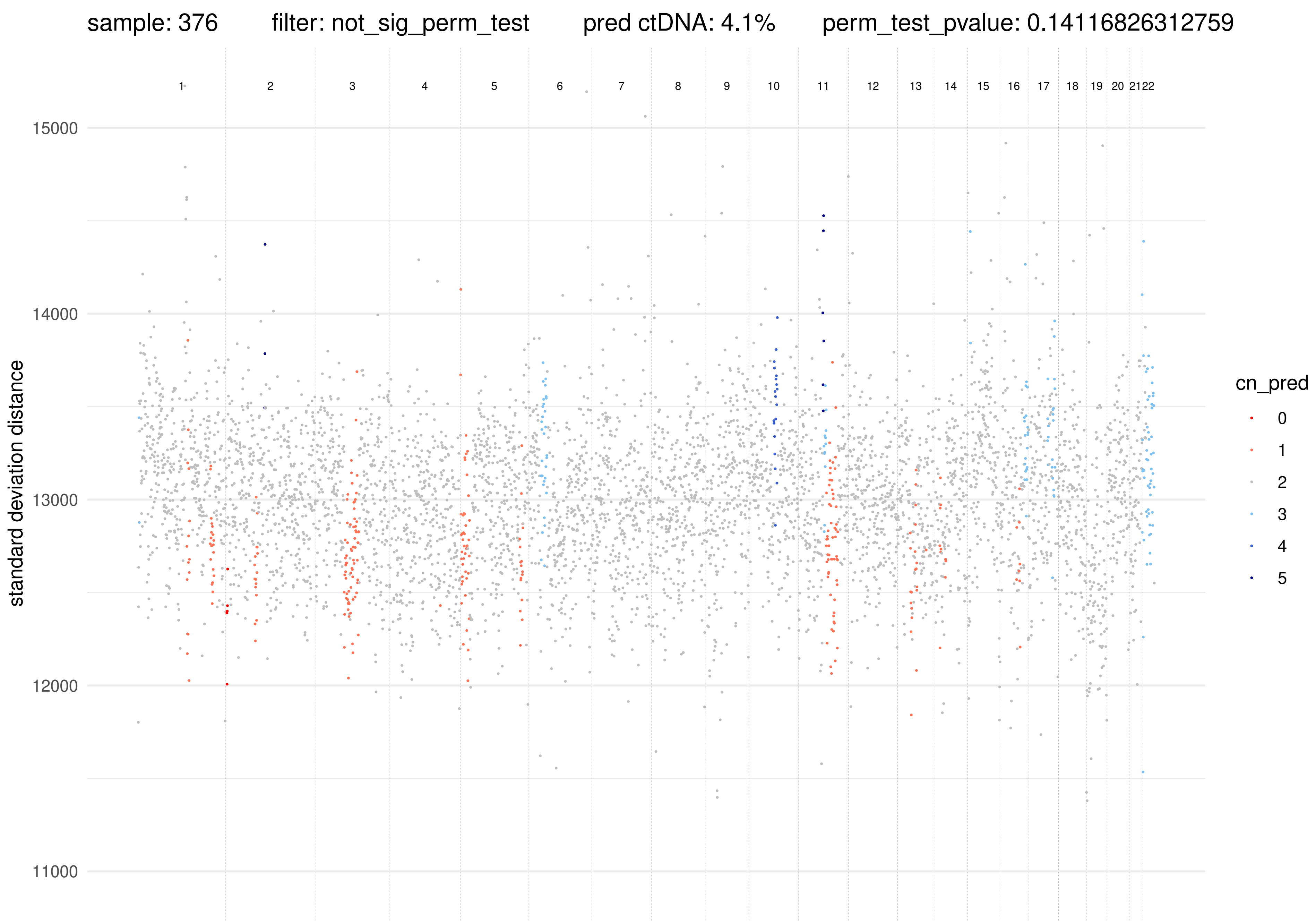

sample: 376
filter: not_sig_perm_test
pred ctDNA: 4.1%
perm_test_pvalue: 0.14116826312759
1
2
3
4
5
6
7
8
9
10
11
12
13
14
15
16
17
18
19
20
21
22
15000
14000
13000
12000
11000
standard deviation distance
cn_pred
0
1
2
3
4
5

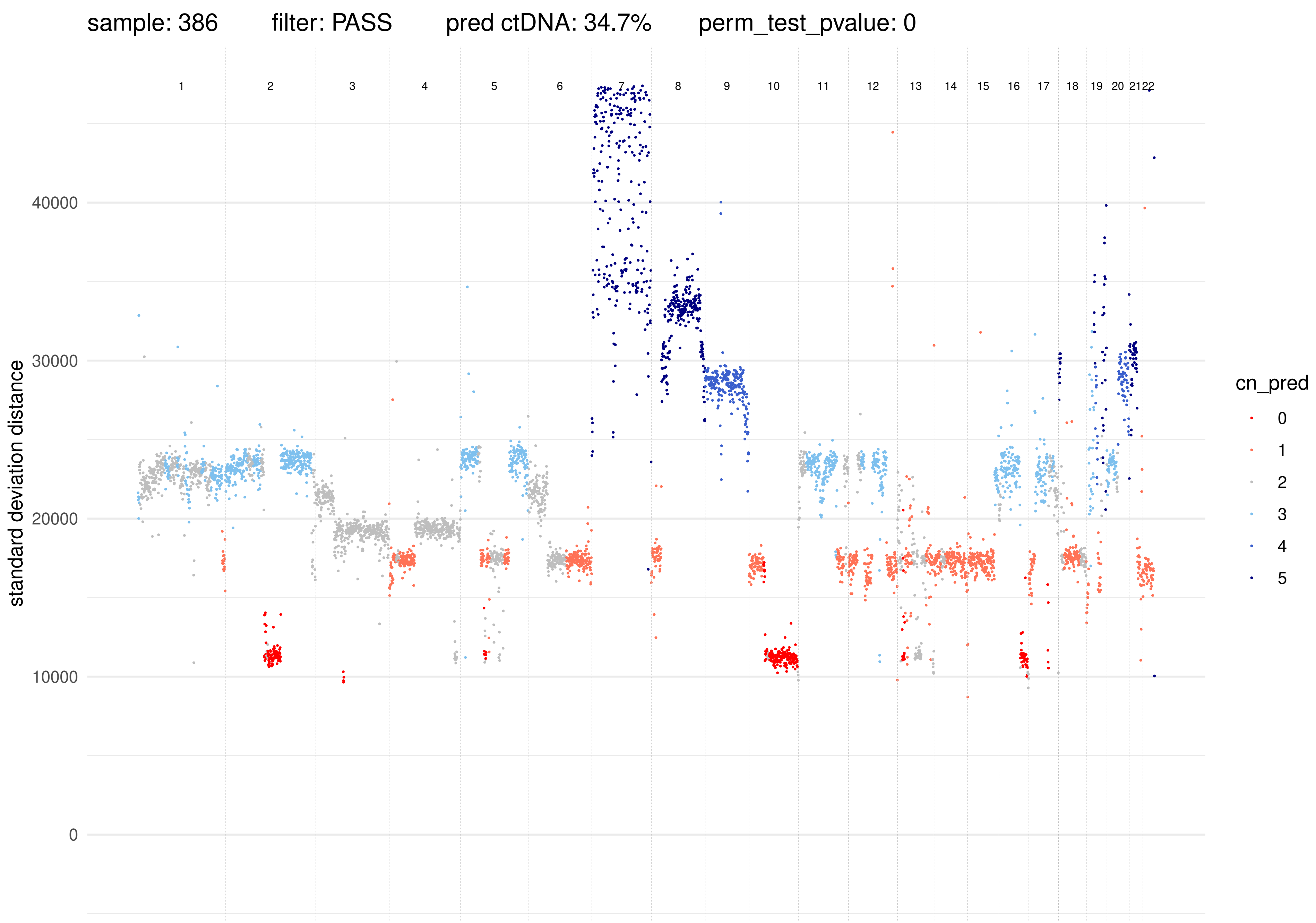
sample: 386
filter: PASS
pred ctDNA: 34.7%
perm_test_pvalue: 0
1
2
3
4
5
6
7
8
9
10
11
12
13
14
15
16
17
18
19
20
21
22
40000
30000
20000
10000
0
standard deviation distance
cn_pred
0
1
2
3
4
5

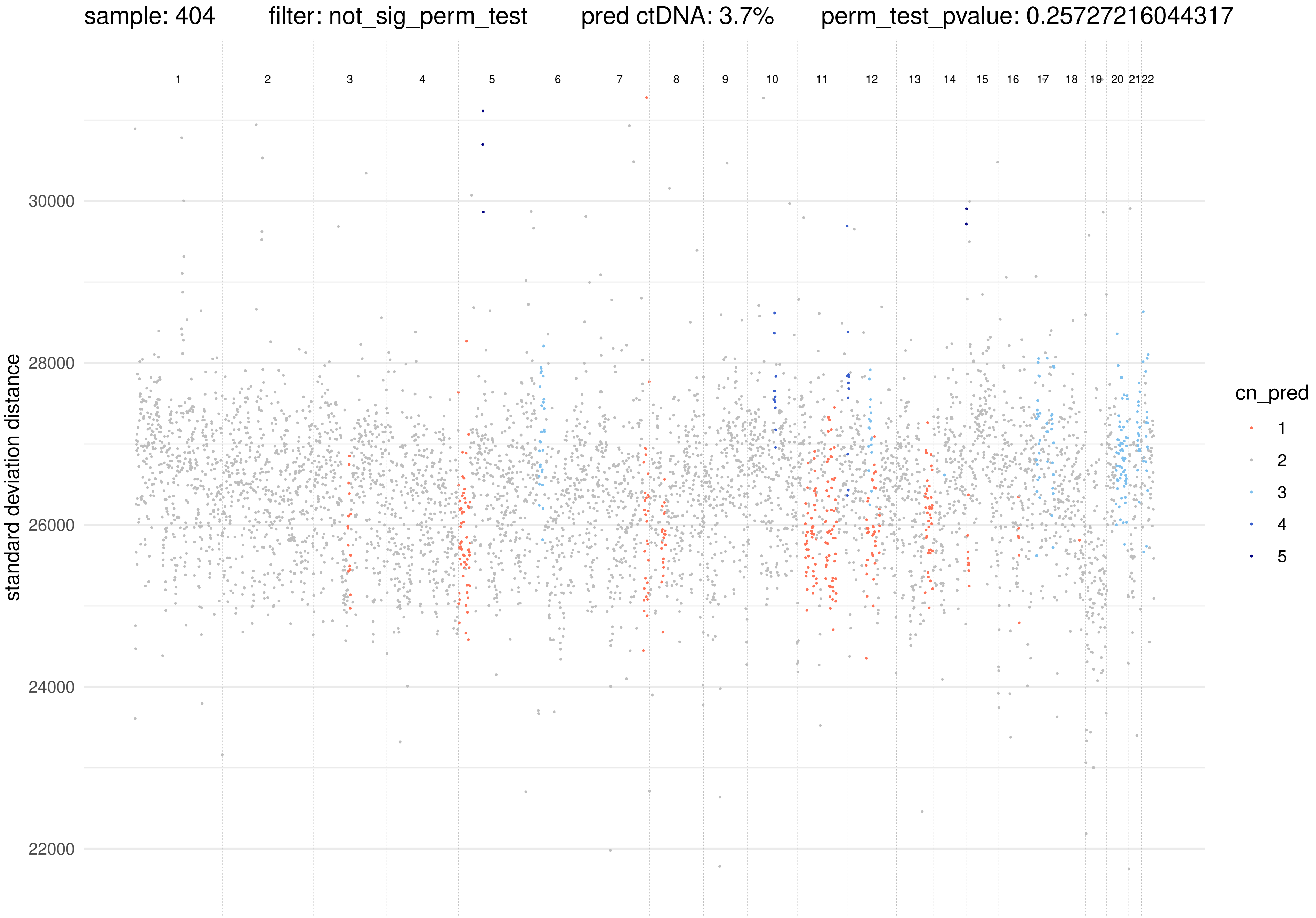

sample: 404
filter: not_sig_perm_test
pred ctDNA: 3.7%
perm_test_pvalue: 0.25727216044317
1
2
3
4
5
6
7
8
9
10
11
12
13
14
15
16
17
18
19
20
21
22
standard deviation distance
30000
28000
26000
24000
22000
cn_pred
1
2
3
4
5

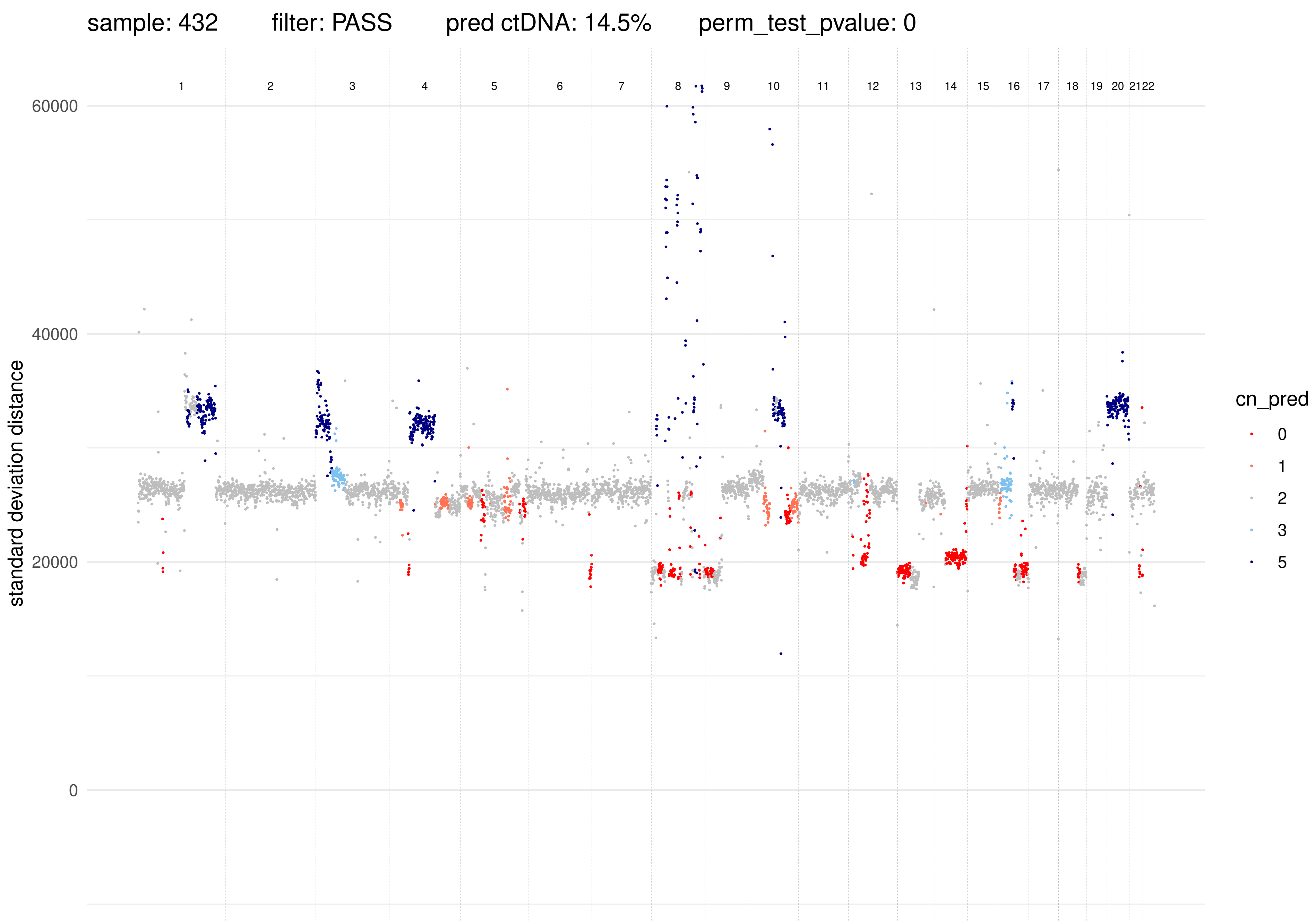

sample: 432
filter: PASS
pred ctDNA: 14.5%
perm_test_pvalue: 0
1
2
3
4
5
6
7
8
9
10
11
12
13
14
15
16
17
18
19
20
21
22
60000
40000
20000
0
standard deviation distance
cn_pred
0
1
2
3
5
